# Critical Investigation of the Usability of Hepatoma Cell Lines HepG2 and Huh7 as Models for the Metabolic Representation of Resectable Hepatocellular Carcinoma

**DOI:** 10.3390/cancers14174227

**Published:** 2022-08-30

**Authors:** Gerda Schicht, Lena Seidemann, Rene Haensel, Daniel Seehofer, Georg Damm

**Affiliations:** 1 Department of Hepatobiliary Surgery and Visceral Transplantation, University Hospital, Leipzig University, 04103 Leipzig, Germany; 2 Saxonian Incubator for Clinical Translation (SIKT), Leipzig University, 04103 Leipzig, Germany

**Keywords:** hepatocellular carcinoma, primary human hepatoma cells, Warburg effect, tumor cell metabolism

## Abstract

**Simple Summary:**

Energy metabolism plays a central role in the liver. Therefore, metabolic alterations in liver cancer are fundamental for the development of diagnostic screening and therapeutic intervention. The aim of our experimental study was to investigate the extent to which commonly used hepatoma cell lines (HCLs) sufficiently represent tumor cells from hepatocellular carcinoma (HCC) from a metabolic point of view. To that end, we successfully established a method for the isolation of primary human hepatoma cells (PHCs). We present the unsurprising finding that cell lines are a poor substitute for primary cells. Surprisingly, our transcript data revealed that malign metabolic adaptions had already occurred in non-tumor-bearing liver tissue of HCC patients. In PHCs, we observed that downregulated metabolic key players showed a correlation with malign transformation and were predominantly pronounced in multilocular HCC. These findings should be taken into account for the future optimization of HCC models for in vitro research.

**Abstract:**

Metabolic alterations in hepatocellular carcinoma (HCC) are fundamental for the development of diagnostic screening and therapeutic intervention since energy metabolism plays a central role in differentiated hepatocytes. In HCC research, hepatoma cell lines (HCLs) like HepG2 and Huh7 cells are still the gold standard. In this study, we characterized the metabolic profiles of primary human hepatoma cells (PHCs), HCLs and primary human hepatocytes (PHHs) to determine their differentiation states. PHCs and PHHs (HCC-PHHs) were isolated from surgical specimens of HCC patients and their energy metabolism was compared to PHHs from non-HCC patients and the HepG2 and Huh7 cells at different levels (transcript, protein, function). Our analyses showed successful isolation of PHCs with a purity of 50–73% (CK18+). The transcript data revealed that changes in mRNA expression levels had already occurred in HCC-PHHs. While many genes were overexpressed in PHCs and HCC-PHHs, the changes were mostly not translated to the protein level. Downregulated metabolic key players of PHCs revealed a correlation with malign transformation and were predominantly pronounced in multilocular HCC. Therefore, HCLs failed to reflect these expression patterns of PHCs at the transcript and protein levels. The metabolic characteristics of PHCs are closer to those of HCC-PHHs than to HCLs. This should be taken into account for future optimized tumor metabolism research.

## 1. Introduction

Liver cancer is the sixth most commonly diagnosed cancer and was the third leading cause of cancer-related death worldwide in 2020 [1]. Hepatocellular carcinoma (HCC) is the most frequent type of primary liver cancer [2]. Curative treatment options for HCC are restricted to hepatic resection or liver transplantation. Hepatic resection, as the first-line therapy, increases the five-year survival rate up to 60–70%, but it can only be offered to patients with limited disease stages who still have good liver function. Since HCC mostly occurs in cirrhotic livers, many HCC patients do not meet these criteria. Liver transplantation has the advantage of removing both the tumor and the damaged liver. However, it is only suitable for selected subgroups of patients with early tumor stages. For cases of advanced or progressive HCC, several systemic treatment options exist, yet they all have unsatisfactory long-term success. Moreover, treatment is often accompanied by toxic side effects [3]. Consequently, the need for novel diagnostic and therapeutic approaches is ongoing.

In HCC, cellular metabolism is altered, favoring anaerobic over aerobic pathways (the Warburg effect). Glucose uptake is enhanced and the shift to anaerobic glycolysis results in an increased production of lactate. Clinically, this is reflected by elevated lactate serum levels in HCC patients [4]. Moreover, the expression of protein coding genes for glycolysis is changed in HCC. Hexokinase isoform 2 (HK2) is upregulated in HCC and is a predictor of reduced survival of HCC patients [5,6]. Additionally, genes involved in fatty acid synthesis, such as acetyl-Co-A carboxylase (ACAC), are upregulated in HCC [7]. However, the dysregulation of metabolic processes enabling tumor cell metastasis and proliferation also provides an opportunity for therapeutic intervention. Signaling pathways can be targeted by inhibitors to prevent tumor growth and disease progression [8].

The gold standard for the in vitro reproduction of human liver function are primary human hepatocytes (PHHs) that are freshly isolated from human liver sections [9]. In Vitro cancer research in the field of HCC is predominantly performed with HCC models based on hepatoma cell lines (HCLs) such as HepG2 and Huh7, both representing dedifferentiated cells [10]. Tissues of HCC patients show high heterogeneity, which cannot be accurately reflected by an HCL generated from a single clone [11]. Moreover, a cell clone always behaves the same way, which does not correspond to the situation in the patient. Thus, insights from experiments with HCLs cannot be directly transferred to the patients. This gap could, however, be bridged by analyses of primary human hepatoma cells (PHCs) that are directly isolated from HCCs. Analogously to PHHs representing the healthy liver, PHCs would provide more realistic insights into the in vivo liver tumor than HCLs do.

Tumor cell isolation typically suffers from contamination with nonparenchymal and immune cells. Known major hepatic contaminants are fibroblasts and cholangiocytes. Cells of hepatocellular origin are characterized by cytokeratin 18 (CK18), whereas CK19 is characteristic of cells of cholangiocellular origin. Additionally, CK19 can be detectable as a biliary and progenitor marker in both PHHs and PHCs [12]. Vimentin as a mesenchymal marker can be expressed by fibroblasts, but it can also be expressed by tumor cells during tumorigenesis [13]. Surface marker TE-7 is used to identify fibroblasts in cell cultures [14]. Alpha-smooth muscle actin (α-SMA)-positive cells represent myofibroblasts, also known as cancer-associated fibroblasts (CAFs) [15]. Additionally, the expression of specific genes can be used for the characterization of PHCs, e.g., glypican-3 (GPC3), serine protease inhib-itor Kazal type 1 (SPINK1), secreted phosphoprotein-1 (SPP1) and karyopherin subunit alpha 2 (KPNA2) [16]. In contrast, fibroblasts are characterized by collagen type I alpha 2 chain (COL1A2), twist family BHLH transcription factor 2 (TWIST2) and fibroblast growth factor 7 (FGF7) [17].

Therefore, the isolation of PHCs required a thorough initial characterization and purity control. The PHCs could then be used in further metabolic investigations and their potential as in vitro HCC models could be determined.

In this study, we aimed to investigate the extent to which commonly used HCLs sufficiently represent HCC cells from a metabolic point of view. We investigated a selected set of genes and proteins to provide a broad overview of metabolic pathways in order to identify the most important ones. As mentioned above, glucose metabolism in particular is altered in tumor cells. Glycogenesis is represented by GSK3A and GSK3B, whereas gluconeogenesis is characterized by FOXO1. Relevant signaling cascades are mapped by AKT in three isoforms (AKT1, AKT2, AKT3) as well as MAPK3 and MAPK1. Representative for glycolysis are HIF1A and PFKL. If glucose deficiency is present in cells, lipogenesis is activated, detected by ACACA and ACACB. Furthermore, the formation of lactate is represented by LDHA, while formation of ketone bodies is characterized by HMGCL and BDH1. We isolated PHCs and characterized their metabolic profiles in comparison to PHHs from the same patients (HCC-PHHs), PHHs from non-HCC patients (non-HCC-PHHs) and the HCLs HepG2 and Huh7.

## 2. Materials and Methods

### 2.1. Tissue

Liver tissues and HCC tumor samples from 14 patients undergoing liver surgery at the Department of Hepatobiliary Surgery and Visceral Transplantation at University Hospital Leipzig, Germany, were included in this study (Table 1). All subjects gave their oral and written informed consent prior to their participation and sample retrieval. This study was conducted in accordance with the Declaration of Helsinki and the protocol was approved by the Ethical Committee at the Medical Faculty, Leipzig University (178/16-lk, 12 July 2016).

### 2.2. Isolation of Primary Liver Cells

#### 2.2.1. Isolation of Primary Human Hepatocytes

Tissue samples for PHH isolation were obtained from macroscopically tumor-free areas of resected livers (Table 1; D1–4 and DH2–10). PHHs were isolated as described previously [19,20,21] by a two-step EGTA/collagenase perfusion technique. Cells were pooled, washed with phosphate-buffered saline (PBS, Gibco, Paisley, UK) and resuspended in PHH culture medium (William’s Medium E, GlutaMAX™ (WME, Gibco, Paisley, UK) supplemented with 10% fetal bovine serum (FBS, Merck Biochrom, Berlin, Germany), 100 U/100 μM penicillin/streptomycin, 1% nonessential amino acids (MEM NEAA 100×), 15 mM HEPES, 1 mM sodium pyruvate (all provided by Gibco, Paisley, UK), 1 μg/mL dexamethasone (Fortecortin^®^, Merck, Darmstadt, Germany) and 80 IU/l human insulin (Lilly Deutschland GmbH, Bad Homburg, Germany/Sanofi-Aventis, Frankfurt am Main, Germany). Cell count and viability were determined using a Neubauer chamber with the trypan blue exclusion technique (Sigma-Aldrich, St. Louis, MO, USA).

#### 2.2.2. Isolation of Primary Human Hepatoma Cells

PHCs were isolated from the tumor tissues of HCC-diagnosed patients (Table 1; DH1–6). The tissue was washed twice with PBS and covered with perfusion solution I (143 mM sodium chloride, 100 mM HEPES (both provided by Carl Roth, Karlsruhe, Germany)), 67 mM potassium chloride, 2.4 mM ethylene glycol-bis(2-aminoethyl ether)-N,N,N′,N′-tetraacetic acid (EGTA), 5 mM N-acetyl-L-cysteine (all provided by Sigma–Aldrich, St. Louis, MO, USA) and minced with a scalpel in a Petri dish. The tissue was transferred to a beaker, covered with perfusion solution I and stirred carefully at 37 °C for 20 min. Afterward, the tissue pieces were allowed to settle for 2 min and the supernatant was discarded. The wash step was repeated a second time. The tumor tissue was digested by adding digestion solution (67 mM sodium chloride, 10 mM HEPES, 6.7 mM potassium chloride, 0.5% bovine serum albumin (BSA), calcium chloride dihydrate (all provided by Sigma–Aldrich, St. Louis, MO, USA), 16.67% DNase I and 100% collagenase P (both obtained from Roche, Basel, Switzerland)) and stirred carefully for 10 min at 37 °C. The digestion process was stopped by transferring the supernatant through cell strainers to 50 mL centrifuge tubes on ice that were previously filled with stop solution consisting of 83.3% PBS and 16.7% FBS. The cell suspension was washed twice with PBS for 10 min at 4 °C and 240× *g*. The supernatant was discarded, and the cells were resuspended in PHC culture medium (DMEM (Gibco, Paisley, UK) supplemented with 10% FBS, 100 U/100 μM penicillin/streptomycin, 1% MEM NEAA 100×, 10 mM HEPES, 1 mM sodium pyruvate and 2 mM L-glutamine). Cell count and viability were determined using a Neubauer chamber with the trypan blue exclusion technique.

### 2.3. Cell Culture and Sampling

After cell isolation, PHHs and PHCs were seeded on rat tail collagen-coated cell culture plates or snap-frozen and stored at −80 °C for later characterization. Collagen from rat tails was prepared in our laboratory according to a protocol described elsewhere [22].

The HCLs HepG2 and Huh7 were used as a reference. For each cell line, cultures of three different passages were used and regarded as independent biological samples. Cell lines were cultured using standard methods (in DMEM supplemented with 10% FBS and 100 U/100 μM penicillin/streptomycin). The medium was changed three times a week and the cells were trypsinized (trypsin 0.25%/EDTA 0.02% in PBS, w/o Ca and Mg, PAN-Biotech GmbH, Aidenbach, Germany) upon reaching 70–80% confluency and replated at a lower density.

Primary cells and cell lines were cultured for 16 h at 37 °C and 5% CO_2_ in the respective culture medium. Following this adherence period, the cells were washed twice with PBS and the medium was changed to the appropriate serum-free medium for 4 h at 37 °C and 5% CO_2_. After this starvation period, cells were harvested with trypsin 0.25%/EDTA 0.02% in PBS, w/o Ca and Mg (PAN-Biotech GmbH, Aidenbach, Germany) for 5 min at 37 °C, 5% CO_2_ or analyzed directly as described below. For functional assays, cell culture supernatants were collected after the 4 h starvation period. Lipid and glycogen measurements reflect the full cultivation period of adherence and the starvation phase of 20 h.

### 2.4. Immunofluorescence Staining

The PHCs and corresponding PHHs from the same donor (HCC-PHHs) were seeded on separate rat tail collagen-coated Cellview cell culture slides (Greiner Bio-One GmbH, Frickenhausen, Germany) at a density of 34 000 cells/cm^2^ in the corresponding cell culture medium. After adherence overnight, the cells were washed twice with PBS for 5 min and fixed with 4% paraformaldehyde solution for 20 min (Carl Roth, Karlsruhe, Germany). Afterward, the cell membranes were permeabilized with 0.2% Triton^™^ X-100 (Sigma–Aldrich, St. Louis, MO, USA) for 10 min at room temperature. Cells were washed three times with PBS for 5 min. Nonspecific binding sites were blocked with 2% BSA solution for 1 h and with human Fc block (Becton Dickinson, Franklin Lakes, NJ, USA) for 10 min. Primary antibodies (Table S1) were diluted in 0.2% BSA solution and incubated overnight. Cells were stained with secondary antibodies for 1 h (Table S1). Cell nuclei were counterstained with Hoechst solution (Hoechst 33342 Solution, Thermo Fisher Scientific, Waltham, MA, USA) and analysis was performed using a laser scanning microscope (LSM 700; Carl Zeiss, Oberkochen, Germany).

### 2.5. RNA Isolation and Quantification

Reverse transcription quantitative real-time PCR (RT–qPCR) experiments were performed according to the recommendations of the MIQE Guidelines [23]. Total RNA was extracted from cultured primary cells and snap-frozen cell pellets using peqGOLD TriFast (VWR International, Radnor, PA, USA) following the manufacturer’s protocol. RNA from cell lines was isolated using an RNeasy^®^ Mini Kit in combination with an RNase-Free Deoxyribonuclease (DNase) Set (Qiagen, Hilden, Germany) according to the manufacturer’s instructions. The purity and integrity of the RNA were determined using a NanoDrop 2000 (Thermo Fisher Scientific, Waltham, MA, USA). RNA was reverse transcribed using a QuantiTect Reverse Transcription Kit (Qiagen, Hilden, Germany) according to the manufacturer’s instructions. Specific primers for the genes listed in Table S2 were commercially sourced from Qiagen. Gene-specific intron-spanning primers for the genes listed in Table 2 were designed with Primer 3 software. Quantification of messenger RNA expression was performed in triplicate with 25 ng cDNA applied to each reaction using the QuantiFast SYBR^®^ Green PCR Kit (Qiagen, Hilden, Germany) with the 7500 Real-Time PCR System and the 7500 Software v2.0.6. (Applied Biosystems, Foster City, CA, USA) according to the manufacturer’s instructions (see Table S3). Glyceraldehyde-3-phosphate dehydrogenase (GAPDH), glucuronidase β (GUSB) and 18S ribosomal RNA (RRN18S) were used as reference genes. Relative gene expression was calculated using the geNorm algorithm [24].

### 2.6. Western Blot Analyses

After culturing or storage at −80 °C, cells were lysed with radioimmunoprecipitation assay (RIPA) buffer (Tris 50 mM (pH 7.4), 150 mM NaCl (both provided by Carl Roth, Karlsruhe, Germany), 1 mM EDTA, 0.5% sodium deoxycholate, 0.5 mM Na_3_VO_4_ (all provided by Sigma–Aldrich, St. Louis, MO, USA), 2.5 mM NaF (Riedel-de-Haen, Seelze, Germany) in dH_2_O mixed with the proteinase inhibitors 0.1% aprotinin, 0.1% 4-(2-aminoethyl)benzolsulfonylfluorid (AEBSF) and 1% Nonidet P-40 (all provided by Sigma–Aldrich, St. Louis, MO, USA)) by mixing and ultrasonic treatment. Protein concentrations were quantified using the BCA assay as described below. Samples were adjusted to protein quantities of 1–2.34 µg/µL with dH_2_O and 4× Laemmli sample buffer (Bio–Rad Laboratories, Inc., Hercules, USA) according to the manufacturer’s instructions and heated for 5 min at 95 °C. For all samples, a total of 15–35 µg protein was separated using 8–15% sodium dodecyl sulfate–polyacrylamide gel electrophoresis (SDS–PAGE) at a voltage of 80 V for 30 min and 100 V for 90 min. Proteins were transferred to nitrocellulose membranes (LI-COR Biosciences, Lincoln, NE, USA) using a tank blot system (Bio–Rad Laboratories, Inc., Hercules, CA, USA) for 17 h at 0.3 A. After immunoblotting, the gels were stained with ROTIPHORESE^®^Blau R (Carl Roth, Karlsruhe, Germany) and analyzed using Fusion Fx 5 to detect any remaining protein in the gels (Vilber Lourmat Deutschland GmbH, Eberhardzell, Germany). The membranes were stained to determine the total protein amount and normalized with Revert™ 700 Total Protein Stain (LI-COR Biosciences, Lincoln, NE, USA) according to the manufacturer’s protocol. Afterward, the membranes were blocked with 1× Intercept^®^ (TBS) Blocking Buffer (LI-COR Biosciences, Lincoln, NE, USA) for 1 h. Primary antibodies (Table S4) were diluted in 1× Intercept^®^ (TBS) Blocking Buffer with 0.1% TWEEN^®^ 20 (Sigma–Aldrich, St. Louis, MO, USA) and the membranes were incubated overnight at 4 °C. For detection, secondary antibodies (Table S4) were used and diluted in 1× Intercept^®^ (TBS) Blocking Buffer with 0.1% TWEEN^®^ 20 and incubated for 1 h. The membranes were dried and the proteins were quantified using an Odyssey 9120 Imaging System (LI-COR Biosciences, Lincoln, NE, USA). Analyses were performed using Image Studio™ Software (LI-COR Biosciences, Lincoln, NE, USA). The protein amount was calculated by normalizing the fluorescent signal to the total protein. To avoid variability between the different blots, the values were normalized to the included positive control.

### 2.7. Functional Assays

#### 2.7.1. Glucose Assay

The high energy demand of cancer cells is reflected in their increased glucose uptake [25]. The glucose consumption of primary cells and cell lines was enzymatically measured in the supernatants by using Fluitest GLU (Analyticon Biotechnologies AG, Lichtenfels, Germany). The difference between the glucose content of the cell culture medium specified by the manufacturer and the measured value allowed us to draw conclusions regarding the formation or consumption of glucose by the cells. A standard curve of D-(+)-glucose (Sigma–Aldrich, St. Louis, MO, USA) was prepared and the assay was performed according to the manufacturer’s instructions. Absorbance was measured at 550 nm with a microplate reader (Synergy H1, BioTek, Winooski, VT, USA).

#### 2.7.2. Glycogen Assay

Hepatocytes store large amounts of glycogen, the cellular storage form of glucose. Glycogen biosynthesis and degradation are two vital mechanisms for the maintenance of normal blood glucose levels. In cancer cells, glycogen metabolism is presumed to be a major energy source [26]. To determine glycogen storage in cells, an amyloglucosidase treatment and a glucose assay, closely following established methods [27], were performed. In brief, glucose was extracted from the cells and eliminated in an alkaline solution before glycogen was hydrolyzed by amyloglucosidase to ß-D-glucose for quantification.

After culturing, the cells were detached, snap-frozen and stored at −80 °C until further analysis. Thawed cells were lysed by sonification. The remaining enzyme function was terminated by adding 7% perchloric acid (Sigma, St. Louis, MO, USA) and the suspension was neutralized by the addition of 0.5 M sodium hydroxide (Carl Roth, Karlsruhe, Germany). Samples were heated to 100 °C for 10 min to release the stored glycogen and remove any free glucose. Then, 2 M acetate buffer (acetic acid and potassium acetate, Carl Roth, Karlsruhe, Germany) containing 4 mg/mL amyloglucosidase was added and the samples were incubated for 2 h at 55 °C. Afterward, a glucose assay was performed as described above.

#### 2.7.3. Pyruvate Assay

Glucose is glycolytically converted to pyruvate, the substrate for further energy production, via oxidative phosphorylation, anaerobic glycolysis or aerobic glycolysis (Warburg effect) [28]. Pyruvate concentration in the cells was determined by a coupled enzyme assay (Pyruvate Assay Kit, Sigma–Aldrich, St. Louis, MO, USA), resulting in a colorimetric product proportional to the pyruvate concentration. The assay was performed according to the manufacturer’s instructions. In brief, cells were detached, washed in PBS, snap-frozen and stored at −80 °C until measurement. After homogenizing the samples in assay buffer, the cells were centrifuged to remove insoluble material. Then, the samples were deproteinized with perchloric acid (Sigma, St. Louis, MO, USA) for 5 min and neutralized by adding potassium hydroxide (Carl Roth, Karlsruhe, Germany). A standard curve was prepared and triplicates of standards and samples were mixed with the reaction mix. After incubating for 30 min, the absorbance was measured at 570 nm with a microplate reader (Synergy H1, BioTek, Winooski, VT, USA).

#### 2.7.4. Lactate Assay

In the presence of oxygen, differentiated cells primarily oxidize pyruvate in the mitochondria, thereby generating CO_2_, H_2_O and adenosine triphosphate (ATP). Larger amounts of lactate are produced under anaerobic conditions. In contrast, the Warburg effect utilized by tumor cells ferments pyruvate to lactate regardless of oxygen availability [28]. Lactate determination was performed using Fluitest La (Analyticon Biotechnologies AG, Lichtenfels, Germany) according to the manufacturer’s instructions. The lactate consumption was measured in cell culture supernatants by determining the difference between the lactate content of the cell culture medium specified by the manufacturer and the measured value. Lactate oxidase cleaves lactate into H_2_O_2_ and pyruvate. H_2_O_2_ reacts in the presence of peroxidase (POD) with 4-aminoantipyrine and TBHB (Tribrom-3-hydroxybenzoic acid) to a red quinoneimine dye. A standard curve was prepared with Lactate IC-Standard-Solution (Carl Roth, Karlsruhe, Germany). Fluitest reaction mix was added to the standard and sample and incubated for 5 min at 37 °C. The absorbance was measured in triplicate at 540 nm with a microplate reader (Synergy H1, BioTek, Winooski, VT, USA).

#### 2.7.5. Lipid Determination (Oil Red O Assay)

Lipids are another means of nutrient storage, and they deliver structural components for cell proliferation. Hepatocellular carcinoma cells exhibit not only a reprogramming of glucose but also of lipid metabolism [29]. To visualize and quantify intracellular lipid contents in the cells, Oil Red O staining was performed. This diazo dye (Sigma–Aldrich, St. Louis, MO, USA) is able to bind to neutral lipids and stains them red. The cells were washed and fixed with Roti^®^-Histofix 4% (Carl Roth, Karlsruhe, Germany) for 30 min. Then, the cells were washed with 60% 2-propanol (Carl Roth, Karlsruhe, Germany), dried and incubated with Oil Red O working solution (0.35% Oil Red O in 2-propanol, diluted in dH_2_O in a 3:2 ratio) for 20 min. The staining solution was discarded and the nonfixed dye was removed by washing three times with dH_2_O. Lipid staining was evaluated using a light microscope (Eclipse TS100, Nikon, Tokyo, Japan) and the cells were then air-dried. The Oil Red O stain was extracted with 100% 2-propanol and the absorbance was measured at 492 nm with a microplate reader (Synergy H1, BioTek, Winooski, VT, USA).

#### 2.7.6. Ketone Body Assay

Under carbohydrate deprivation, hepatocytes and cancer cells break down acetyl Co-A from fatty acid oxidation to generate ketone bodies as alternative energy metabolites [29]. To detect ketone bodies in the samples, a colorimetric assay for measuring β-hydroxybutyrate (β-HB) levels was used (β-Hydroxybutyrate Colorimetric Assay Kit, Cayman Chemical, Ann Arbor, MI, USA). To detect the amount of β-HB in the cells, the oxidation of D-3-hydroxybutyrate to acetoacetate by the enzyme 3-hydroxybutyrate dehydrogenase was measured. Thereby, NAD^+^ is reduced to NADH which reacts with the colorimetric detector water-soluble tetrazolium 1 (WST-1) and produces a formazan dye. The cells were harvested after culturing using cell scrapers and the assay was performed according to the manufacturer’s protocol. In brief, cells were lysed in assay buffer and sonicated. Cell pellets were stored at −80 °C until analysis. A standard curve was prepared, and samples were measured in triplicate. A developing solution was added to the standard and samples and incubated at room temperature for 25 min. Absorbance was measured at 450 nm with a microplate reader (Synergy H1, BioTek, Winooski, VT, USA).

#### 2.7.7. Protein Determination (Bicinchoninic Acid Assay)

For normalization purposes, the cellular protein amount was determined using a bicinchoninic acid assay (BCA). Cells were lysed with RIPA buffer and ultrasonicated. A standard curve based on BSA was used and BCA reaction mix (Sigma–Aldrich, St. Louis, MO, USA) was added to the samples according to the manufacturer’s instructions. After incubation for 30 min at 37 °C in the dark, the absorbance was measured at 550 nm with a microplate reader (Synergy H1, BioTek, Winooski, VT, USA).

#### 2.7.8. Quantification and Statistical Analyses

**Figure 1:**
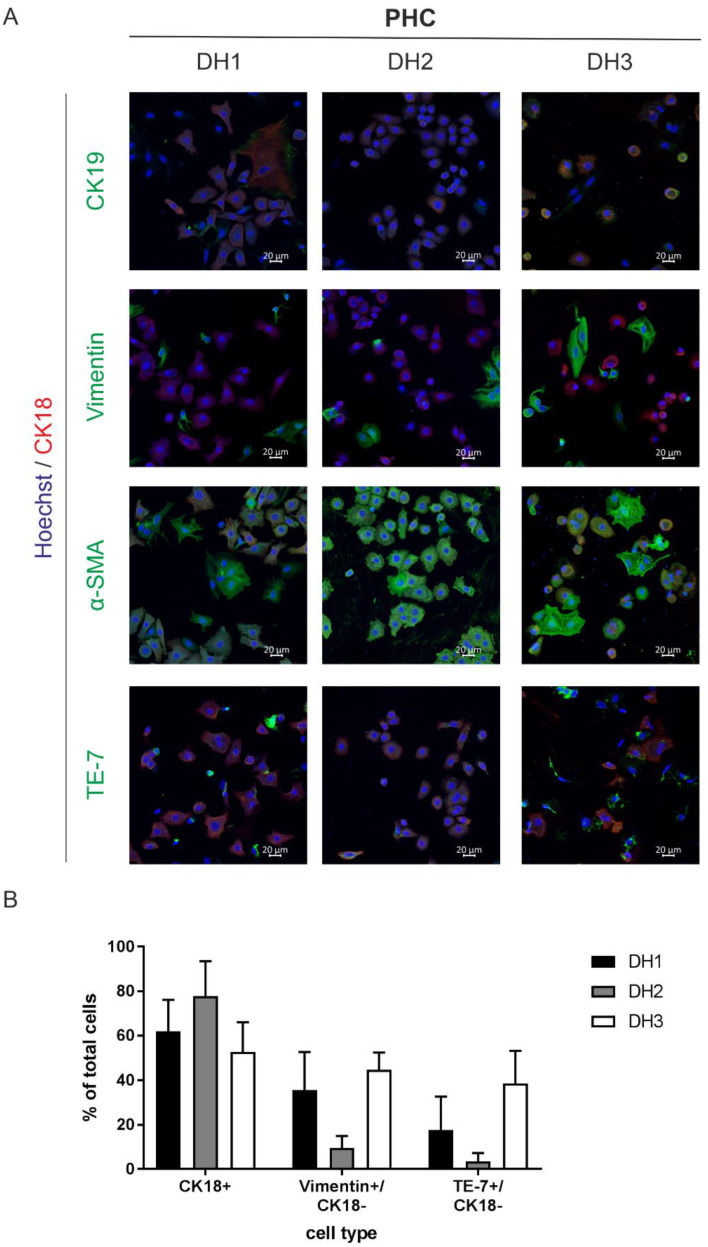
Characterization of the attached cell fractions isolated from HCC tissue samples. (**A**) All primary tumor cells were nuclei-stained with Hoechst (blue) and CK18 (red) as a marker for cells of hepatocellular origin. Additionally, cells were stained with a biliary/progenitor or tumor stem cell marker (CK19), an early differentiating epithelial cell marker (vimentin), a marker for liver fibrosis progression (α-SMA) and a fibroblast marker (TE-7, all marked with green). Cells were analyzed after overnight adherence by immunofluorescence staining and laser scanning microscopy at 20× magnification. The scale bar is 20 µm. (**B**) Immunofluorescence staining of cells of donor DH1-DH3 were used for marker quantification. Five randomly taken images per well were acquired at 20× magnification using laser scanning microscopy, and the cells were counted manually based on their specific staining. Total cells were counted by Hoechst nuclei staining. Data are shown as means + SD (N = 1, n = 5). Abbreviations: PHC, primary human hepatoma cell; CK, cytokeratin; α-SMA, alpha-smooth muscle actin; TE-7, antifibroblast antibody clone.

**Figure 2:**
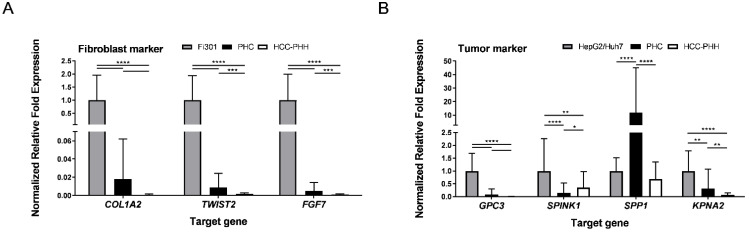
Characterization of isolated primary cells by genetic markers. HCC-PHHs (N = 8) and PHCs (N = 6) were analyzed after cell isolation using RT-qPCR. Relative mRNA expression levels of (**A**) fibroblast markers (COL1A2, TWIST2, FGF7) and (**B**) tumor markers (GPC3, SPINK1, SPP1, KPNA2) were determined. Fi301 (N = 3) and HepG2/Huh7 (N = 6) cells were used as positive controls. Values are means + SD, n = 3, two-way ANOVA and post hoc Šidák correction or Tukey’s test, statistical analyses were conducted on ΔC_T_ values, * *p* ≤ 0.05, ** *p* ≤ 0.01, *** *p* ≤ 0.001, **** *p* < 0.0001. Abbreviations: PHC, primary human hepatoma cell; HCC, hepatocellular carcinoma; PHH, primary human hepatocyte; COL1A2, collagen type I alpha 2 chain; TWIST2, twist family BHLH transcription factor 2; FGF7, fibroblast growth factor 7; GPC3, glypican-3; SPINK1, serine protease inhibitor Kazal type 1; SPP1, secreted phosphoprotein-1; KPNA2, karyopherin subunit alpha 2.

**Figure 3:**
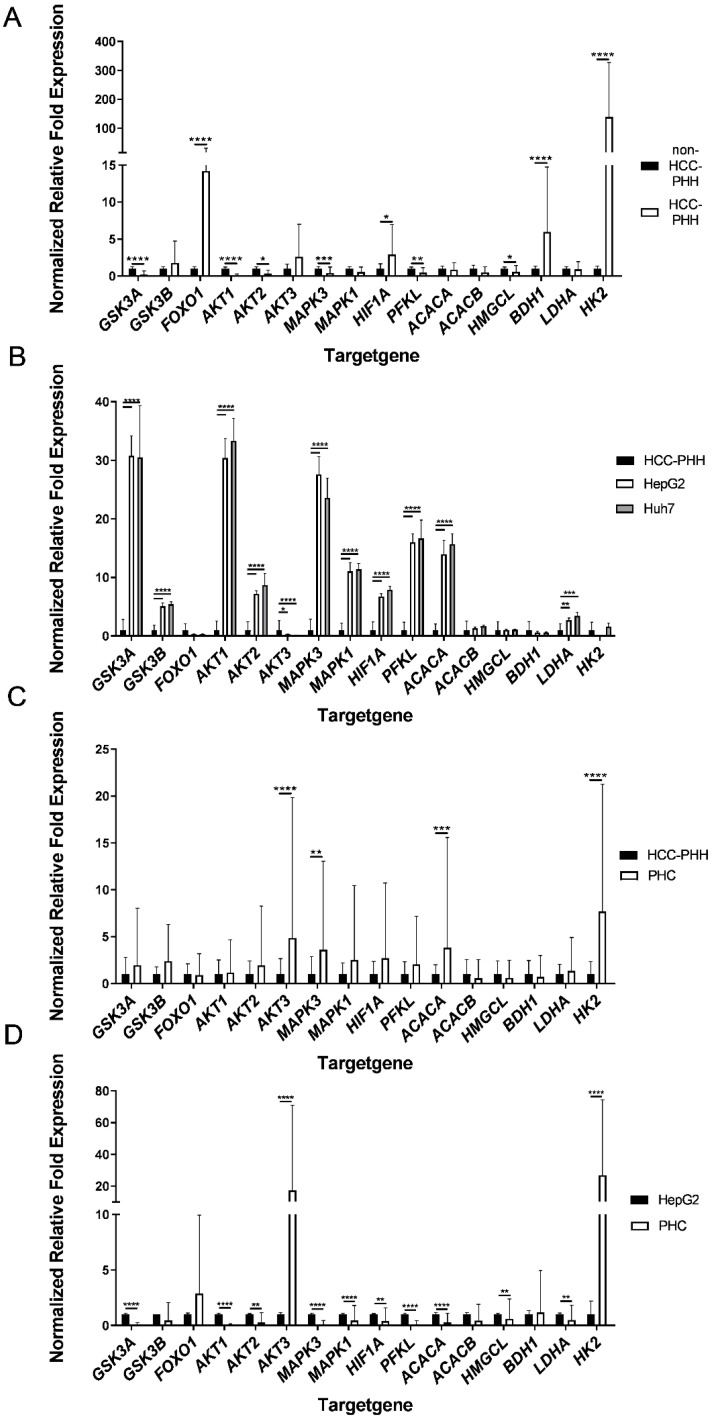
Differentially expressed energy metabolism genes of hepatocytes, hepatocellular carcinoma cells and hepatoma cell lines. Cells were cultured for 20 h or snap-frozen directly after isolation, and relative mRNA expression levels of central actors in hepatic energy metabolism were determined by RT-qPCR. (**A**) PHHs from non-HCC patients (N = 4) were compared with PHHs from HCC-diagnosed patients (N = 9) and showed distinct metabolic gene expression profiles. (**B**) Gene expression data of the hepatoma cell lines HepG2 and Huh7 (N = 3) were normalized to PHHs from HCC donors (N = 9). (**C**) PHCs (N = 6) showed different gene expression patterns to PHHs and (**D**) to HepG2 cells (N = 3). Data are shown as means + SD, n = 3, two-way ANOVA and post hoc Šidák correction, statistical analyses were conducted on ΔC_T_ values, * *p* ≤ 0.05, ** *p* ≤ 0.01, *** *p* ≤ 0.001, **** *p* < 0.0001. Abbreviations: PHH, primary human hepatocyte; PHC, primary human hepatoma cell; HCC, hepatocellular carcinoma; GSK3A, glycogen synthase kinase 3 alpha; GSK3B, glycogen synthase kinase 3 beta; FOXO1, forkhead box O1; AKT1, AKT serine/threonine kinase 1; AKT2, AKT serine/threonine kinase 2; AKT3, AKT serine/threonine kinase 3; MAPK3, mitogen-activated protein kinase 3; MAPK1, mitogen-activated protein kinase 1; HIF1A, hypoxia inducible factor 1 alpha; PFKL, phosphofructokinase liver type; ACACA, acetyl-CoA carboxylase alpha; ACACB, acetyl-CoA carboxylase beta; HMGCL, 3-hydroxymethyl-3-methylglutaryl-CoA lyase; BDH1, 3-hydroxybutyrate dehydrogenase 1; LDHA, lactate dehydrogenase A; HK2, hexokinase 2.

**Figure 4:**
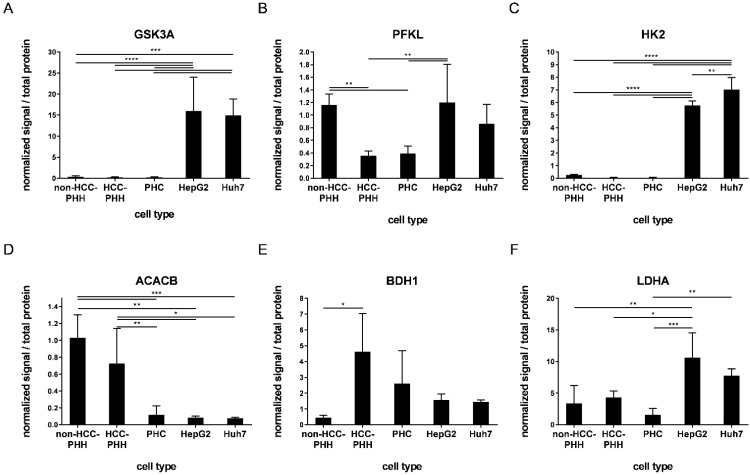
Expression of proteins related to energy metabolism. Cells were cultured for 20 h or snap-frozen directly after isolation, and protein expression levels of central actors in hepatocyte metabolism were determined by Western blot analysis. (**A**–**F**) PHHs from non-HCC patients (non-HCC-PHH, N = 4), PHHs from HCC-diagnosed patients (N = 5) and PHCs (N = 6) were compared with the hepatoma cell lines HepG2 and Huh7 (N = 3) concerning their expression levels of metabolic proteins. Data are shown as means + SD and were normalized to total protein amount and positive control, n = 1–2, one-way ANOVA and post hoc Tukey’s test, * *p* ≤ 0.05, ** *p* ≤ 0.01, *** *p* ≤ 0.001, **** *p* < 0.0001. Abbreviations: PHH, primary human hepatocyte; PHC, primary human hepatoma cell; HCC, hepatocellular carcinoma; GSK3A, glycogen synthase kinase 3 alpha; PFKL, phosphofructokinase liver type; HK2, hexokinase 2; ACACB, acetyl-CoA carboxylase beta; BDH1, 3-hydroxybutyrate dehydrogenase 1; LDHA, lactate dehydrogenase A.

**Figure 5:**
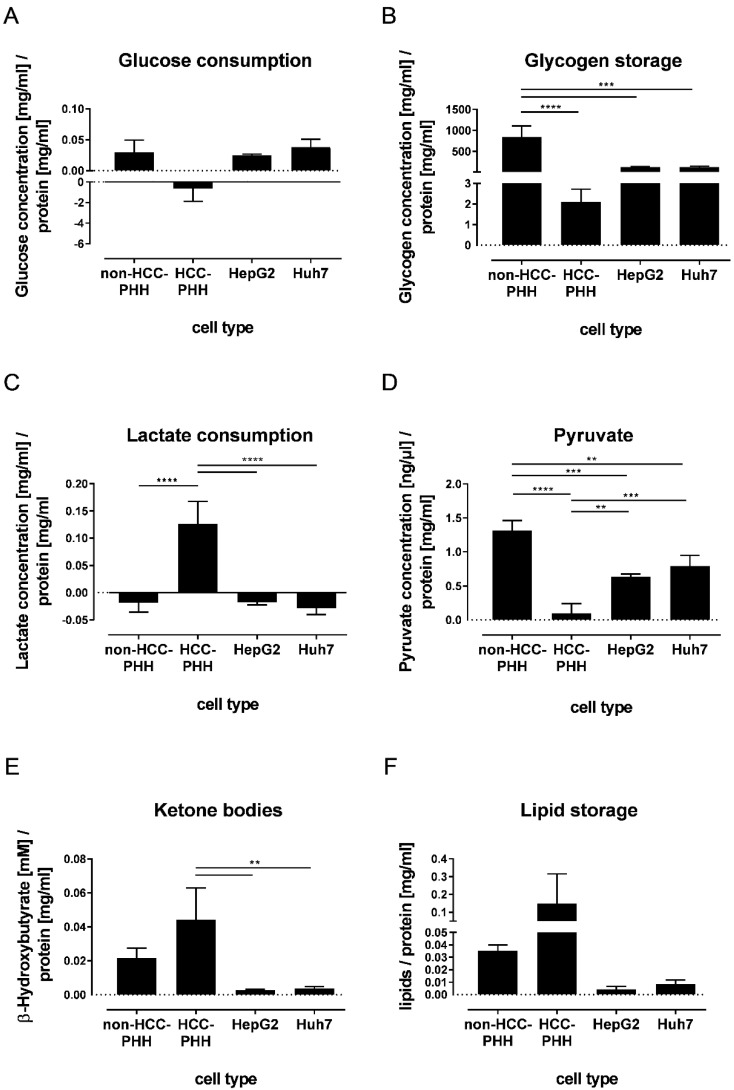
Dependence of cell functionality on cell type and differentiation state. Cells were cultured for 20 h and metabolic functionality was determined using functional assays. (**A**–**F**) Healthy hepatocytes (non-HCC-PHH, N = 4) and PHHs from HCC-diagnosed patients (N = 4) were compared with hepatoma cell lines HepG2 and Huh7 (N = 3). Data are shown as means + SD and were normalized to protein content, n = 3, one-way ANOVA and post hoc Tukey’s test, ** *p* ≤ 0.01, *** *p* ≤ 0.001, **** *p* < 0.0001. Abbreviations: PHH, primary human hepatocyte; PHC, primary human hepatoma cell; HCC, hepatocellular carcinoma.

**Figure 6:**
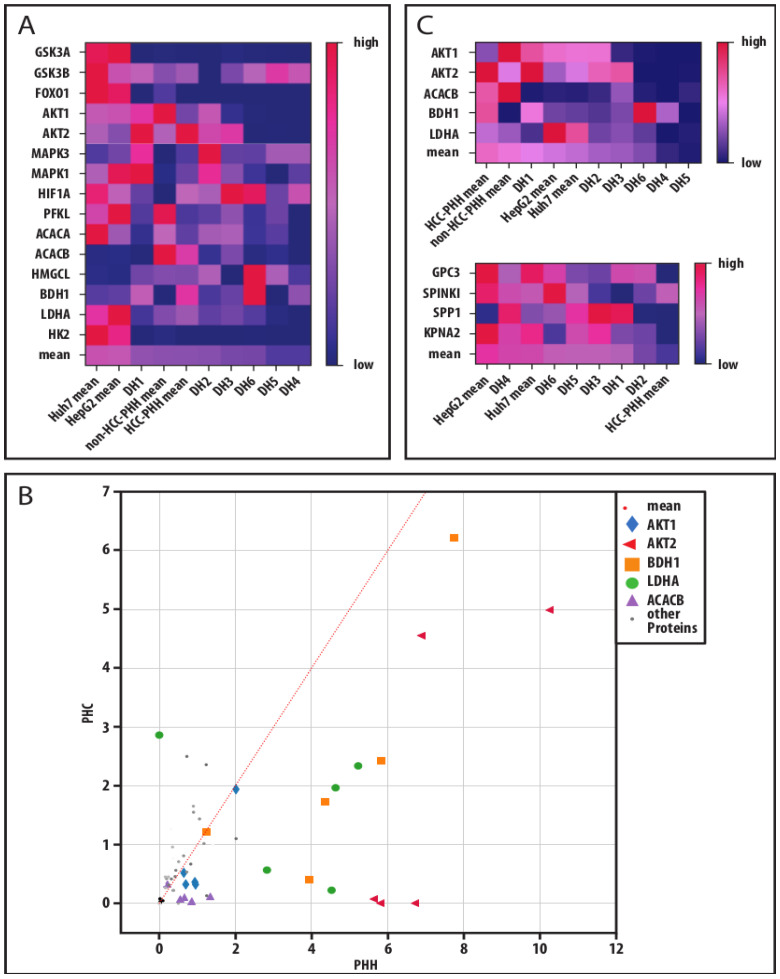
Metabolic and cancerous dedifferentiation of primary human hepatoma cells. (**A**) Absolute protein values of measured markers were transferred to a relative percentage scale. The relative values were displayed in their respective color on a color gradient. Mean values of a donor or cell entity were calculated from all markers in a column and the columns were sorted by their respective relative mean values resulting in heat maps. This analysis was performed for all investigated metabolic protein markers to display their metabolic dedifferentiation. (**B**) Absolute protein values of metabolic markers measured in PHCs, and their corresponding PHHs were also plotted to display their metabolic shift through cancerous dedifferentiation and key players were identified by their distance to the identity function (slope = 1). (**C**) For investigation of a link between metabolic and cancerous dedifferentiation in PHCs derived from different donors compared to hepatoma cell lines and PHHs, the heat maps of the selected metabolic key players and of hepatocellular tumor markers are displayed. Abbreviations: PHH, primary human hepatocyte; HCC, hepatocellular carcinoma; DH, HCC-diagnosed patient 1–6; GSK3A, glycogen synthase kinase 3 alpha; GSK3B, glycogen synthase kinase 3 beta; FOXO1, forkhead box O1; AKT1, AKT serine/threonine kinase 1; AKT2, AKT serine/threonine kinase 2; MAPK3, mitogen-activated protein kinase 3; MAPK1, mitogen-activated protein kinase 1; HIF1A, hypoxia inducible factor 1 alpha; PFKL, phosphofructokinase liver type; ACACA, acetyl-CoA carboxylase alpha; ACACB, acetyl-CoA carboxylase beta; HMGCL, 3-hydroxymethyl-3-methylglutaryl-CoA lyase; BDH1, 3-hydroxybutyrate dehydrogenase 1; LDHA, lactate dehydrogenase A; HK2, hexokinase 2; GPC3, glypican-3; SPINK1, serine protease inhibitor Kazal type 1; SPP1, secreted phosphoprotein-1; KPNA2, karyopherin subunit alpha 2.

Experiments were performed with different quantities of biological replicates (either different donors in case of primary cells or different passages in case of cell lines), as indicated in the figures. GraphPad Prism 7 software (San Diego, CA, USA) was used for the chart design (Figure 1, Figure 2, Figure 3, Figure 4 and Figure 5) and statistical analyses. Outliers were identified via the ROUT test (aggressiveness 1%). All data are presented as the means of biological replicates ± standard deviations (SD). To compare multiple groups, one-way and two-way ANOVA were performed followed by a post hoc Šidák correction or Tukey’s test. Statistical significance was considered at * *p* ≤ 0.05, ** *p* ≤ 0.01, *** *p* ≤ 0.001, **** *p* < 0.0001. Figure 6 was created using the open access software tool Python 3.8.

## 3. Results

### 3.1. Characterization of the Isolated Primary Human Hepatoma Cells Revealed Mixed Populations with a High Yield of Cancer Cells

We established a method for the isolation of liver tumor cells, allowing for the isolation of PHCs, corresponding CAFs and nonparenchymal liver cells (NPCs). The cell suspensions were characterized for cell entities and their purity using immunofluorescence staining and RT–qPCR (see Table 1, DH1–DH6). Immunofluorescence staining required the attachment of the cells, which was possible in three out of six cell suspensions isolated from HCC-diagnosed patients (see Table 1, DH1–DH3).

The immunofluorescence staining targeted specific surface markers of the parenchyme and NPCs, allowing us to discriminate cells of hepatocellular differentiation (CK18) from cells of cholangiocellular differentiation (CK19) and fibroblasts (TE-7, α-SMA, vimentin). Immunofluorescence staining revealed that all three cell cultures were mixtures of PHCs and fibroblasts (Figure 1A). In addition to the cells that were single positive for one of the fibroblast or cholangiocellular markers, we observed coexpression of fibroblast and cholangiocellular markers with the hepatocellular marker CK18. The amount of PHCs, which were unambiguously identified by CK18, ranged between 50 and 73% (Figure 1B). Coexpression with CK18 was not observed for vimentin and was negligible for TE-7 (Figure S1B). The determination of potential fibroblast contamination revealed 10–45% and 3–39% for the expression of vimentin and TE-7, respectively (Figure 1B). α-SMA showed similar expression to vimentin and TE-7 in CK18-negative cells (7–42%, Figure S1A), but it was also expressed in 100% of CK18+ cells (Figure S1B). Thus, it cannot be considered a reliable marker for fibroblast contamination. The expression of CK19 alone ranged between 8 and 18%, suggesting minor contamination with cholangiocytes (Figure S1A). Additionally, we detected coexpression of CK19 with CK18, which is an indicator of PHCs with progenitor cell characteristics (Figure S1B).

In summary, the characterization of PHCs using immunofluorescence staining provided a very good overview of potential contaminations from the tumor microenvironment. However, this method was limited to adherent PHC isolates and limited to only about half of the cell samples examined.

Freshly isolated cells were additionally characterized for specific mRNA expression of tumor (GPC3, SPINK1, SPP1, KPNA2) and fibroblast (COL1A2, TWIST2, FGF7) marker genes using RT–qPCR (Figure 2). Representative tumor cell lines (HepG2, Huh7) and fibroblast cell lines (Fi301) were used as positive controls. Messenger RNA expression of the analyzed fibroblast markers was markedly lower in PHCs and available HCC-PHHs than in Fi301 cells (Figure 2A, Table S5), indicating that the relative amount of fibroblasts after cell isolation was lower than the immunofluorescence analysis of adherently cultured cells implied.

All four analyzed HCC markers were expressed in PHC isolates (Figure 2B). With the exception of SPP1, mRNA expression levels were lower in PHCs and HCC-PHHs than in the HCLs. While GPC3 and KPNA2 were hardly detectable in HCC-PHHs, SPINK1 showed an even higher expression level in HCC-PHHs than in PHCs (Table S6). Notably, the tumor marker expression levels of PHCs and HCC-PHHs from different donors showed marked interindividual variations (see Figure S2).

In summary, cell suspensions isolated from HCC tissues contained at least 50% PHCs but also a considerable number of fibroblasts and were slightly contaminated with cholangiocytes. This should be considered in further data interpretation.

### 3.2. Primary Human Hepatoma Cells Showed a Metabolic Shift in Energy Metabolism Genes

It has been reported that metabolic adaptations play a crucial role in HCC. Therefore, the expression of genes encoding proteins related to metabolism was examined by RT–qPCR in the hepatoma cell lines HepG2 and Huh7 and in the PHCs and PHHs derived from HCC or non-HCC patients.

These studies revealed major alterations in the expression of energy metabolism genes in HCC-PHHs and non-HCC-PHHs (Figure 3A). The genes for GSK3A, AKT1, AKT2, MAPK3, PFKL and HMGCL were expressed at significantly lower levels in HCC-PHHs. In contrast, FOXO1, HIF1A, BDH1 and HK2 showed striking overexpression in HCC-PHHs. HK2 was even 138-fold overexpressed in HCC-PHHs (Table S7).

The comparison of gene expression in HepG2 and Huh7 cells with that in HCC-PHHs revealed that the majority of the genes showed significantly higher expression in the HCLs (Figure 3B, Tables S8 and S9). Both HCLs displayed similar gene expression levels.

The direct comparison of PHCs and PHHs showed highly varying alterations in their metabolic gene profile (Figure 3C). The four genes—AKT3, MAPK3, ACACA and HK2—showed a striking upregulation in the analyzed tumor cell samples. All of the other genes showed no significant expression differences between HCC-PHHs and PHCs (Table S10). These findings indicate a metabolic shift [30] from hepatocytes to tumor cells based on dedifferentiation processes.

The hepatoma cell line HepG2 and PHCs showed few similarities in gene expression levels (Figure 3D). Striking overexpression of AKT3 and HK2 was observed in PHCs. In contrast, GSK3A, AKT1, AKT2, MAPK3, MAPK1, HIF1A, PFKL, ACACA, HMGCL and LDHA showed higher expressions in HepG2 cells (Table S11). Except for HMGCL, this corresponds to the upregulated gene expression in the HCLs relative to the HCC-PHHs (Figure 3B). The expression levels of metabolic genes in Huh7 cells were comparable to those in HepG2 cells (see Figure S3). In summary, the HCLs HepG2 and Huh7 do not sufficiently represent the metabolic gene expression profiles of PHCs from surgical HCC patients.

### 3.3. Effects of Cell Types on the Expression Levels of Metabolic Proteins

To validate the gene expression data and to further investigate the differences between HCLs and PHCs from patient tumors, the protein levels of the above mentioned metabolic targets were investigated using western blot analysis (see Figure S5). The most relevant alterations between HCLs and PHCs included GSK3A, PFKL, HK2, ACACB, BDH1 and LDHA (Figure 4), whereas all other investigated proteins showed fewer alterations (Figure S4).

In general, except for HK2 and ACACA, HepG2 and Huh7 cells displayed no significant differences in protein expression. HK2, GSK3A and PFKL are major players in glucose metabolism and they regulate the intracellular availability of glucose, storage and glycolysis. The expression levels of these enzymes were equally low in PHCs and HCC-PHHs, whereas their expression in HepG2 cells was significantly upregulated (Figure 4A–C, Tables S12–S14). Except for PFKL, this was also the case for the Huh7 cell line. Additionally, PFKL showed lower expression in PHCs and HCC-PHHs than in non-HCC-PHHs. The ACACB enzyme is responsible for rate-limiting steps in fatty acid synthesis and was highly expressed in all PHHs. The expression was strongly decreased in PHCs and HCLs (Figure 4D, Table S15). BDH1 catalyzes the interconversion of acetoacetate and (R)-3-hydroxybutyrate, the two major ketone bodies produced during fatty acid catabolism. PHHs from HCC patients showed significantly higher expression in comparison to non-HCC-PHHs, suggesting a fasting state in HCC neighboring liver tissue. PHCs expressed BDH1 levels comparable to those of HCC-PHHs (Figure 4E, Table S16). LDHA, catalyzing the conversion of pyruvate to lactate, showed a significant difference between HepG2 cells and primary cells, while Huh7 cells only differed from PHCs (Figure 4F, Table S17).

Notably, the high levels of AKT3 and HK2 transcripts found in PHCs and HCC-PHHs could not be confirmed at the protein level (Figure 4C and Figure S4C). While AKT3 protein was not detectable at all in the investigated samples, HK2 protein was significantly lower in PHCs than in HCLs. In contrast, FOXO1 was expressed at the protein level in non-HCC-PHHs and both cell lines but was absent in PHCs and HCC-PHHs. The general expression pattern of FOXO1 was comparable to that of the other glycolytic enzymes described above (Figure S4D). Taken together, the protein data confirmed the majority of metabolic differences as indicated by the transcript data.

### 3.4. Major Metabolic Changes Arise at a Premalignant Stage in the Diseased Liver

The adaptation of various metabolic proteins in the disease progression of HCC gives rise to the question of whether and when these adaptations manifest on the functional level. Therefore, we investigated changes in the metabolite concentrations of central metabolic pathways using biochemical assays. Due to their limited adherence and low cell numbers, this analysis was not performable with PHCs. Glycolysis was determined by measuring the glucose content in the cell culture supernatant, allowing the discrimination between glucose consumption and glucose formation (Figure 5A). HCLs HepG2 and Huh7, as well as non-HCC PHHs, showed a consumption of glucose from the cell culture medium. In contrast, the HCC-PHHs produced glucose during the cultivation time (Table S18).

Glycogen, as a storage form of glucose, was detected in non-HCC-PHHs (Figure 5B). These cells showed significantly higher glycogen levels than all other cell types. No significant differences in glycogen storage between HCLs and HCC-PHHs could be determined (Table S19).

Lactate production occurs in the fasting state and is a known feature of tumor cells. This was confirmed for the hepatoma cell lines HepG2 and Huh7, which both showed a slight production of lactate. In our experimental setup, non-HCC-PHHs also produced lactate, in accordance with LDHA expression. In contrast, the HCC-PHHs clearly consumed lactate (Figure 5C, Table S20).

Pyruvate was investigated as a further central metabolite in energy metabolism (Figure 5D). Again, the proportion of pyruvate in non-HCC-PHHs was the highest. In contrast, the pyruvate amount was significantly decreased in HCC-PHHs compared with non-HCC-PHHs and cell lines (Table S21).

Ketone bodies are metabolites that are produced during fasting. The amount of ketone bodies was significantly increased in HCC-PHHs compared to HCLs (Figure 5E). This finding can be explained by the increased BDH1 expression at the protein level. No differences between HCC-PHHs and non-HCC-PHHs were observed (Table S22).

The storage of lipids in liver cells was investigated as they make an essential contribution to energy metabolism. The lipid accumulation varied widely in HCC-PHHs and was higher by trend than in non-HCC-PHHs and HCLs, in line with ACACB expression (Figure 5F, Table S23). In accordance with these findings, the histopathological donor data show steatosis in only 5–15% of the hepatocytes from non-HCC donors and in up to 60% of the hepatocytes from HCC donors (Table 1).

Taken together, the functional data confirm metabolic changes which derived from protein expression data. Notably, HCC-PHHs showed a distinct pattern of central processes in energy metabolism compared to non-HCC-PHHs but also to HCLs, revealing that the metabolic alteration of HCC already takes place at a premalignant stage.

### 3.5. Global Data Analysis Shows Diverging Traits of Metabolic and Cancerous Dedifferentiation

To take individual donor variations into account, we performed a global analysis of our analyzed protein markers for the investigation of a cancer-related adaptation of hepatic energy metabolism at progressive levels of dedifferentiation (Figure 6). For that, we displayed our individual donor data in heatmaps. A mean for all markers was calculated and the samples were ranked accordingly. In both analyses, the HCLs showed the highest values, representing their predominantly high expression levels (Figure 6A,C). PHCs from donors DH4–DH6 were ranked on the low expression end of the scale. All three donors had multilocular HCC and therefore more advanced disease stages despite their low histological gradings. The expression of a marker in PHCs was compared to its expression in the corresponding PHHs of the same donor and displayed as coordinates for each sample (Figure 6B). The analysis revealed that five markers (AKT1, AKT2, BDH1, LDHA and ACACB) were expressed at significantly lower levels in PHCs than in PHHs. These markers were chosen to describe metabolic dedifferentiation. Again, the PHCs from multilocular HCCs (DH4–DH6) showed the lowest protein expressions. PHHs showed the highest protein expressions, while the HCLs were situated between the PHHs and most of the PHCs. In comparison, a similar ranking analysis performed on the tumor marker RT-qPCR data revealed the highest mean expressions in HCLs and the lowest in PHHs, with most of the PHCs in between. In line with the metabolic dedifferentiation ranking, the three donors with multilocular HCCs (DH4–DH6) showed similar expression levels and clustered together with the HCLs. In contrast, the donors DH1-DH3 clustered close to the PHHs. These were also the samples showing adherence abilities, in contrast to DH4–DH6.

Taken together, our global data analysis showed that downregulated metabolic key players correlated with malignant transformation and were predominantly pronounced in multilocular HCC.

## 4. Discussion

For meeting the needs of developing new diagnostic methods and treatment strategies, the usage of HCLs as an HCC model is widely accepted. Additionally, HCC tissue samples collected from resected liver tissues of HCC-diagnosed patients serve as frequent materials in HCC research. The aim of our study was to examine whether HCLs adequately represent the characteristics of HCC in patients who qualify for curative surgical therapy.

We successfully established a method allowing the isolation of PHCs from HCC tissue samples with viabilities between 60% and 93%. Functional cell cultures were obtained from only 50% of the investigated liver cell fractions. The loss of the capability to adhere to collagen-coated cell culture plastics suggests an altered expression of cell–matrix interaction proteins [31]. It is known that the integrin pattern of hepatoma cells and extracellular matrix (ECM) in HCC is changed [32]. Additionally, hepatoma cells can run into an AMPK-mediated energy crisis when detached from their in vivo ECM during the isolation process, resulting in nonadherent dead cells [33,34]. Therefore, PHCs showing low attachment capacities are highly dependent on their tumor environment and are much more differentiated than autarchic HCLs. However, fibroblasts, which are a known part of tumor tissue, show inherently good adherence capacities. Subsequent cell characterization revealed varying purities depending on the characterization technique used. Using immunofluorescence on adherent cells, 50–73% CK18+ PHCs were identified. Using RT–qPCR for purity analysis, we were able to characterize the full amount of isolated cell fractions. In contrast to the analysis of adherent cells, the investigation of fibroblastic markers revealed less fibroblast contamination of the tumor cell fraction. Therefore, we assume a low presence of fibroblasts in our PHC isolations that must be taken into account when interpreting any experimental results.

For further characterization of PHCs, the expression of the following HCC-specific tumor markers was investigated: GPC3, SPINK1, SPP1 and KPNA2. GPC3 is characterized as a developmental marker, and SPINK1 is localized downstream of CDH17 (fetal cadherin)/ß catenin. SPP1 and KPNA2 are described as markers linked to rapid disease progression, accompanied by larger tumors or increased AFP levels [35,36]. Thus, these markers target fetal reprogramming (GPC3 and SPINK) and large and fast-growing tumors (KPNA2 and SPP1). Of all analyzed tumor markers, GPC3 showed the lowest expression in PHCs. However, it was the only tumor marker completely absent in PHHs from the same patients. These results are in line with clinical data showing that GPC3 is a very specific hepatic tumor marker that is not expressed in healthy liver tissue and remains absent in liver pathologies such as NAFLD or cirrhosis [37]. Interestingly, SPINK1 was overexpressed in HCC-PHHs rather than in PHCs and therefore was not suitable as a marker to differentiate hepatoma cells from their neighboring liver cells. However, overexpression of SPINK1 is a sign of increased cell migration and invasion in vitro [38]. Therefore, high SPINK1 expression in PHHs could be an early sign of tumorigenesis in diseased livers. Both RNAs of SPINK1 and SPP1 are expressed in cholangiocytes [39], so contamination of our PHCs and HCC-PHHs with cholangiocytes could affect their significance as markers. SPP1 is also described as one of the upregulated signature genes in HCC [35]. It is considered a driver of tumor evolution [40] and its protein osteopontin is a prospective biomarker for HCC. Higher serum levels of osteopontin have already been observed in premalignant chronic liver diseases, and in HCC patients it correlates with poor outcomes [41]. In line with these reports, in our study, SPP1 showed the highest gene expression in PHCs, and its gene expression was also present in HCC-PHHs. SPINK1 and KPNA2, which represent advanced HCC markers, were expressed in low numbers in all cells of the analyzed tissues, which indicates tumors that are not advanced. These tumor characteristic data represent early HCC tumors and are in line with the potential characteristics of resectable HCC. In contrast, the HCLs showed higher expression of all investigated tumor markers in comparison to PHCs and HCC-PHHs, except for SPP1. Taken together, in comparison to HCLs, the analyzed tumor markers were expressed to a much lower extent and highly heterogeneously in PHCs.

Energy metabolism in tumors is often shifted from aerobic to anaerobic metabolism, the so-called Warburg effect. This adaptation is accompanied by increased glucose uptake and lactate production. Our characterization of key players in the energy metabolism of hepatic cells confirmed extensive alterations of these targets in PHCs in comparison to hepatocytes but also to the standard HCLs HepG2 and Huh7. At the transcript level, HK2 was significantly overexpressed in PHCs compared to HCC-PHHs and HCLs. HK2 is highly overexpressed under hypoxic conditions by HIF1A activation [42]. Moreover, HK2 binds to mitochondria, suppressing apoptosis by binding to voltage-dependent anion-selective channel 1 (VDAC1) [43]. Therefore, high HK2 expression in HCC patients correlates with poor overall survival [44]. Our results show highly elevated expression levels of HK2 and HIF1A in PHCs and HCC-PHHs in comparison to non-HCC-PHHs, indicating a hypoxic (peri-)tumor environment. Hypoxia can lead to the expression of the developmental marker GPC3 [45], which is also an enhancer of HIF1A expression [45]. As a consequence of hypoxic conditions, anaerobic metabolism takes place, which was observed by an upregulation of LDHA enzyme only in the HCLs. LDHA is also regulated by HIF1A and GPC3 [42]. This metabolic shift is linked to increased lactate production and a higher uptake of glucose leading to an enhancement of the Warburg effect [46]. In line with the observed LDHA upregulation, the HCLs showed this metabolic shift in our functional assays. In contrast, LDHA expression in PHCs and HCC-PHHs was low, as confirmed by the lactate consumption of HCC-PHHs. Additionally, in HCC-PHHs, low pyruvate and production of glucose were observed in our functional analyses. The observed lactate consumption in conjunction with glucose production could point to a highly active Cori cycle in HCC-PHHs, in which lactate is utilized for gluconeogenesis. However, HCC-PHHs do not seem to store this produced glucose, since their glycogen storage was rather low. We therefore hypothesize that HCC-PHHs might function as feeder cells for their neighboring tumor tissue. Further studies are needed to examine this assumption. HMGCL and BDH1 are both central enzymes in the formation of ketone bodies. HMGCL is downregulated in PHCs and HCC-PHHs in comparison to non-HCC-PHHs. In HCC, hepatocytes can undergo a shift from ketogenesis to ketone oxidation accompanied by the activation of BDH1 and succinyl-CoA:3-ketoacid-coenzyme A transferase (SCOT) [47]. Under serum-starved conditions, hepatoma cells are able to express BDH1 and 3-oxoacid CoA-transferase 1 (OXCT1) as well as oxidize ketones [48]. In PHCs and HCC-PHHs, increased expression was observed for BDH1. At the protein level, its expression varied but it was correlated with the transcript data. BDH1, which catalyzes a reversible reaction in both ketogenesis and ketolysis, is also upregulated under serum starvation conditions [48]. Consequently, our data indicate a high fasting state in HCC and HCC-diseased tissue leading to a consumption of ketone bodies. HCC-PHHs are also effective ketone body producers, as confirmed by metabolite analysis. Furthermore, the lipid content in HCC-PHHs was higher by trend than in non-HCC-PHHs, which reflects the donor data mentioned in Table 1. The majority of the HCC-PHH donors utilized for the functional analyses (DH3-DH6) have higher BMIs, and the histological analyses of their liver tissues revealed a higher grade of steatosis than the tissues from non-HCC-PHH donors. Ketone bodies are therefore likely produced via fatty acid beta oxidation. The high lactate content in HCC-PHHs presumably reflects a metabolic shift toward the Warburg effect that already takes place in the tumor neighboring hepatic cells. In summary, our results suggest an early metabolic shift using lactate and ketone bodies as alternative fuels—not only in HCCs but also in adjacent tissue—as an early sign of tumorigenesis.

The AKT signaling pathway acts as a key regulator of hepatic glucose metabolism, controlling the downstream targets FOXO1 (gluconeogenesis) and GSK3 (glycogen synthesis). AKT1 and AKT2 are downregulated in PHCs and HCC-PHHs. Again, this shows a discrepancy with the HCLs that overexpress AKT1 and AKT2. AKT3 was significantly overexpressed in PHCs compared to hepatocytes and cell lines. Yang et al. reported [49] that AKT3 is upregulated in HCC and HCLs as a result of downregulation of microRNA miR-424. Additionally, it was shown that AKT3 plays a prominent role in embryonic stem cells [50], suggesting that the overexpression of AKT3 could also be associated with dedifferentiation into a progenitor cell or stem cell characteristic of HCC. FOXO1 shows significant overexpression at the transcript level in PHCs and HCC-PHHs in comparison to non-HCC-PHHs. This suggests that tumorigenesis is already taking place in hepatocytes [51]. GSK3A protein expression showed differences between HCLs and primary liver cells. On the transcript level, GSK3B expression was increased in PHCs and cell lines in comparison to non-HCC-PHHs. Neither the FOXO1 nor GSK3 expression levels suggest an influence on energy metabolism, favoring their role in intracellular signaling pathways relevant for HCC development, which is still controversial [52]. In our experiments, MAPK, as a key player in cell proliferation, showed the highest mRNA expression levels in the HCLs. At the protein level, MAPK3 was overexpressed in PHCs compared to PHHs, while the expression of MAPK1 was highest in HepG2 cells. The upregulation of genes in the MAPK pathway and, in particular, downstream signaling pathways has been described as leading to cell proliferation, survival, differentiation and migration [42]. The synthesis of fatty acids in HCC tissue has been described as increased, resulting from upregulation of genes such as acetyl-CoA carboxylase [53]. This is also consistent with our findings that ACACA gene expression in PHCs is significantly increased compared to HCC-PHHs, and even higher expression was seen in HCLs. At the protein level, PHHs showed the highest levels of ACACB compared to PHCs and HCLs. From a purely functional point of view, however, the lipid content was clearly increased in PHCs. Our data are in line with results showing high free fatty acid uptake and ß-oxidation rates in HCC, indicating high lipid metabolism [54].

Our global analysis of metabolic protein profiles at the individual donor levels revealed five proteins with significantly different expression levels in PHCs relative to their corresponding PHHs. Again, the outstanding markers were characteristic of fasting, anaerobic metabolism and shifts in lipid metabolism. Although the individual patterns of metabolic adaptations were heterogeneous, our results are in line with previous reports [54,55] These adaptations were accompanied by a change in the AKT isoform expression pattern. Expression of the central signaling molecules AKT1 and AKT2 was decreased in PHCs in comparison to their corresponding PHHs and thus included in our marker set describing metabolic dedifferentiation. In three donors, AKT2 was completely downregulated in PHCs. Remarkably, these were (again) the three donors with multilocular HCCs (DH4–DH6). Despite their multilocularity, two of these tumors had a histological G1 grading and only one tumor was classified as G2. Hepatic AKT1/2 inhibition and especially AKT2 deletion promote hepatocarcinogenesis [56]. In line with these findings, the ranking of the mean tumor marker expression profiles revealed a clustering of the DH4–DH6 PHCs at the high-expression end of the scale together with the HCLs. The higher expression of the metabolic proteins seen for the HCLs, on the other hand, reflects their metabolic profile that shows traits but not the full aspects of cancerous dedifferentiation as seen in vivo. Presumably, the cell culture conditions for the HCLs did not reflect fasting and anaerobic conditions similar to the in vivo HCC environment.

In summary, the established isolation method allowed the isolation of PHCs in a moderate number. The cell yield of isolated PHCs depends primarily on the available tumor tissue sample. In the majority, the yields were sufficient for a detailed characterization at the transcript and protein levels but were insufficient for a characterization at the functional level. The latter required a culture of attached PHCs, which was only obtained in limited cases. Therefore, our data on PHC characterization are limited to the expression of single proteins in metabolic networks and lack validation at the functional level within these metabolic pathways. A drawback of our functional characterization is that its early time point overlapping with in vitro cell regeneration also affects cellular metabolism. However, PHH cultivation for a longer time leads to dedifferentiation which would impair the comparability with the initially obtained expression data [21].

Our results indicate that PHCs represent tumors of therapeutically relevant HCC candidates that are much better models than the hepatoma cell lines HepG2 and Huh7. This is in contrast to previously published studies. Nwosu et al. [57] showed that the metabolic gene expression pattern of hepatic cancer cell lines well represents those of tumor tissue. However, in their study, they only compared tumor tissue with cell lines (e.g., Huh7, HLE) but not with primary tumor cells. In our study, no primary cells were obtained from histologically dedifferentiated tumors since surgical removal is rarely an option in those cases. Compared with the commonly used cell lines, the PHCs in our study showed a hypoxic and fasting metabolic phenotype. To display this, HepG2 and Huh7 would need to be cultured under low-oxygen and/or -glucose concentration conditions. Therefore, without advanced culturing conditions, HCLs are only partially representative of HCC, which needs to be considered when carrying out research.

Finally, primary liver cells directly derived from HCCs should be preferred over HCLs or at least used for validation of the results obtained from experiments with HCLs. Furthermore, our results indicate that the histological grading into G1 and G2 tumors does not cover the whole picture of dedifferentiation in HCCs.

To date, no staging system considers the tumor metabolome, although this could be a promising tool to improve the diagnosis, prognosis and therapy of HCC patients and to investigate new biomarkers [58]. 

## 5. Conclusions

Early HCC is mostly characterized by well-differentiated tumors classified by histological grading as G1 and G2 tumors. However, research in this field is primarily performed with HCC models based on the HepG2 and Huh7 hepatoma cell lines. Our metabolic investigations confirmed that these hepatoma cell lines are unable to represent early, differentiated HCC tumors. Additionally, the large metabolic alterations in PHHs from HCC patients suggest that the metabolic switch in tumor cells can already be observed in precancerous stages.

## Figures and Tables

**Table 1:** Donor specifications for tissue samples used for primary cell isolation. List of donor number, age, sex, diagnosis and characteristics. Patients are grouped according to diagnosis (non-HCC (D1–4) and HCC (DH1–10)).

Donor	Age	Sex	Diagnosis	BMI	Steatosis [%]	ASH	NASH	Fibrosis	Cirrhosis
D1	74	male	CRLM *	23.6	10	no	no	yes	no
D2	39	male	CRLM *	27.8	15	no	no	yes	no
D3	46	female	CRLM *	20.3	no	no	no	yes	no
D4	28	female	Adenoma	27.5	5	no	no	yes	no
DH1	66	male	HCC G1	34.5	10–60	no	yes	no	yes
DH2	63	male	HCC G1	33.6	5	no	no	yes	no
DH3	79	female	HCC G1	28	60	yes	no	no	yes
DH4	66	male	HCC G1	28	5	no	no	yes	no
DH5	63	male	HCC G1	25	60	no	yes	no	yes
DH6	79	male	HCC G2	27	20	no	yes	yes	no
DH7	76	male	HCC G2	24.8	25	yes	no	yes	yes
DH8	71	male	HCC G1	26.1	20	no	yes	yes	yes
DH9	50	female	HCC G2	62.1	35	no	yes	yes	no
DH10	67	female	HCC G1	43.9	60	no	yes	no	yes

* Patient has received chemotherapy. Abbreviations: CRLM, colorectal liver metastasis; BMI, body mass index; ASH, alcoholic steatohepatitis; NASH, nonalcoholic steatohepatitis; HCC, hepatocellular carcinoma; G1, G2, histological grading according to Hamilton and Aaltonen [18].

**Table 2:** Primer for RT-qPCR.

Gene Name	Type	Primer Sequence
FGF7	forward reverse	GAAGGAGGGGATATAAGAGTGAG ATTCTTCATCTCTTGGGTCCC
GPC3	forward reverse	TGTGCCCATTCTCAACAACG AGCAAAGGGTGTCGTTTTCC
HK2	forward reverse	TACCTGGGTGAGATTGTCCG CAAGCCCTAAGTGTTGCAGG
KPNA2	forward reverse	AGGAAAACCGCAACAACCAG TTTCGGAATCAAACCAGCCC
SPINK1	forward reverse	AGAGACGTGGTAAGTGCGG ATTTGGCCTCTCTTCCCAGG
SPP1	forward reverse	CACACATGGAAAGCGAGGAG TGGAATTCACGGCTGACTTTG
TWIST2	forward reverse	CTACAGCAAGAAGTCGAGCG CTTGCTCAGCTTGTCAGAGG

## Data Availability

The data presented in this study are available on request from the corresponding author.

## Supplementary Materials

The following supporting information can be downloaded at: https://www.mdpi.com/article/10.3390/cancers14174227/s1, Table S1: Antibodies for immunofluorescence. Table S2: Gene specific primers commercially purchased from Qiagen (Hilden, Germany). Table S3: RT-qPCR cycling conditions. Table S4: Antibodies for Western blotting. Table S5: Values of test statistics of fibroblast marker (Figure 2A). Table S6: Values of test statistics of tumor marker (Figure 2B). Table S7: Values of test statistics of RT-qPCR analyses of non-HCC-PHHs and HCC-PHHs (Figure 3A). Table S8: Values of test statistics of RT-qPCR analyses of HCC-PHHs and HepG2 cells (Figure 3B). Table S9: Values of test statistics of RT-qPCR analyses of HCC-PHHs and Huh7 cells (Figure 3B). Table S10: Values of test statistics of RT-qPCR analyses of HCC-PHHs and PHCs (Figure 3C). Table S11: Values of test statistics of RT-qPCR analyses of HepG2 cells and PHCs (Figure 3D). Table S12: Values of test statistics of protein expression analyses of GSK3A (Figure 4A). Table S13: Values of test statistics of protein expression analyses of PFKL (Figure 4B). Table S14: Values of test statistics of protein expression analyses of HK2 (Figure 4C). Table S15: Values of test statistics of protein expression analyses of ACACB (Figure 4D). Table S16: Values of test statistics of protein expression analyses of BDH1 (Figure 4E). Table S17: Values of test statistics of protein expression analyses of LDHA (Figure 4F). Table S18: Values of test statistics of glucose consumption analyses (Figure 5A). Table S19: Values of test statistics of glycogen storage analyses (Figure 5B). Table S20: Values of test statistics of lactate consumption analyses (Figure 5C). Table S21: Values of test statistics of pyruvate analyses (Figure 5D). Table S22: Values of test statistics of ketone bodies analyses (Figure 5E). Table S23: Values of test statistics of lipid storage analyses (Figure 5F). Figure S1: Quantitative evaluation of PHC immunofluorescence staining. Figure S2: Expression pattern of tumor markers in individual donors. Figure S3: Differentially expressed energy metabolism genes of hepatocellular carcinoma and hepatoma cells. Figure S4: Quantitative evaluation of metabolic target proteins. Figure S5: Western Blots of analyzed proteins.
