# Supplementary material for: Critical Investigation of the Usability of Hepatoma Cell Lines HepG2 and Huh7 as Models for the Metabolic Representation of Resectable Hepatocellular Carcinoma
Source: Cancers (Basel). 2022 Aug 30;14(17):4227. doi: 10.3390/cancers14174227 (PMC9454736; doi:10.3390/cancers14174227)
Supplement: Supplementary file 1 [file cancers-14-04227-s001.zip › cancers-1858328-supplementary.pdf]

cancers

MDPI

Supplementary Materials

Critical investigation on the usability of hepatoma cell lines HepG2 and Huh7 as models for the metabolic representation of resectable hepatocellular carcinoma

Gerda Schicht[1,2†], Lena Seidemann[1,2†], Rene Haensel[2], Daniel Seehofer[1,2] and Georg Damm[1,2*]

[1] Department of Hepatobiliary Surgery and Visceral Transplantation, University Hospital, Leipzig University, 04103 Leipzig, Germany
[2] Saxonian Incubator for Clinical Translation (SIKT), Leipzig University, 04103 Leipzig, Germany
* Correspondence: Georg.Damm@medizin.uni-leipzig.de
† These authors contributed equally to this work.

Table S1. Antibodies for Immunofluorescence.

Product name	Type	Host	Dilution	Product Number	Supplier
anti-Cytokeratin 18	Primary antibody	Rabbit	1:150	ab52948	Abcam, Cambridge, UK
anti-Cytokeratin 19	Primary antibody	Mouse	1:1000	ab7754	Abcam, Cambridge, UK
anti-Vimentin	Primary antibody	Mouse	1:500	sc-373717	Santa Cruz Biotechnology, Dallas, USA
anti-Fibroblasts, clone TE-7	Primary antibody	Mouse	1:200	CBL271	Sigma-Aldrich, St. Louis, USA
anti-Actin, α-Smooth Muscle	Primary antibody	Mouse	1:400	A2547-2ML	Sigma-Aldrich, St. Louis, USA
Donkey F(ab')2 Anti-Mouse IgG H&L (Alexa Fluor® 488)	Secondary antibody	Mouse	1:1000	ab181289	Abcam, Cambridge, UK
Donkey F(ab')2 Anti-Rabbit IgG H&L (Alexa Fluor® 647)	Secondary antibody	Rabbit	1:1000	ab181347	Abcam, Cambridge, UK

Table S2. Gene specific primers commercially purchased from Qiagen (Hilden, Germany).

Gene Name	Article Description	Article Number
ACACA	Hs_ACACA_2_SG QuantiTect® Primer Assay	QT01670053
ACACB	Hs_ACACB_1_SG QuantiTect® Primer Assay	QT00996352
AKT1	Hs_AKT1_1_SG QuantiTect® Primer Assay	QT00085379
AKT2	Hs_AKT2_1_SG QuantiTect® Primer Assay	QT00085001
AKT3	Hs_AKT3_1_SG QuantiTect® Primer Assay	QT00082138
BDH1	Hs_BDH1_1_SG QuantiTect® Primer Assay	QT01673525
FOXO1	Hs_FOXO1_1_SG QuantiTect® Primer Assay	QT00044247
GAPDH	Hs_GAPDH_vb.1_SG QuantiTect® Primer Assay	QT02504278
GSK3A	Hs_GSK3A_1_SG QuantiTect® Primer Assay	QT00075306
GSK3B	Hs_GSK3B_1_SG QuantiTect® Primer Assay	QT00057134
GUSB	Hs_GUSB_1_SG QuantiTect® Primer Assay	QT00046046
HIF1A	Hs_HIF1A_1_SG QuantiTect® Primer Assay	QT00083664

Gene Name	Article Description	Article Number
HMGCL	Hs_HMGCL_1_SG QuantiTect® Primer Assay	QT00088921
LDHA	Hs_LDHA_1_SG QuantiTect® Primer Assay	QT00001687
MAPK1	Hs_MAPK1_1_SG QuantiTect® Primer Assay	QT00065933
MAPK3	Hs_MAPK3_3_SG QuantiTect® Primer Assay	QT02589321
PFKL	Hs_PFKL_1_SG QuantiTect® Primer Assay	QT00044814
RRN18S	Hs_RRN18S_1_SG QuantiTect® Primer Assay	QT00199367

Table S3. RT-qPCR cycling conditions.

Step	Time	Temperature
PCR initial heat activation	5 min	95°C
Denaturation	10 s	95°C
Combined annealing/extension	30 s	60°C
Number of cycles	35-40 s	

Table S4. Antibodies for Western blotting.

Protein	Type	Host	Dilution	Product Number	Supplier
ACACA	Primary antibody	Rabbit	1:1000	4190S	Cell Signaling Technology, Inc., Danvers, USA
ACACB	Primary antibody	Rabbit	1:1000	8578S	Cell Signaling Technology, Inc., Danvers, USA
AKT1	Primary antibody	Rabbit	1:1000	75692S	Cell Signaling Technology, Inc., Danvers, USA
AKT2	Primary antibody	Rabbit	1:1000	3063S	Cell Signaling Technology, Inc., Danvers, USA
AKT3	Primary antibody	Rabbit	1:1000	3788S	Cell Signaling Technology, Inc., Danvers, USA
BDH1	Primary antibody	Mouse	1:1000	MA5-15594	Invitrogen, Carlsbad, USA
ERK1/MAPK3	Primary antibody	Mouse	1:1000	MA5-15896	Invitrogen, Carlsbad, USA
ERK2/MAPK1	Primary antibody	Rabbit	1:1000	PA5-17710	Invitrogen, Carlsbad, USA
FOXO1	Primary antibody	Rabbit	1:1000	9454S	Cell Signaling Technology, Inc., Danvers, USA
GSK3A	Primary antibody	Rabbit	1:1000	4337S	Cell Signaling Technology, Inc., Danvers, USA
GSK3B	Primary antibody	Mouse	1:1000	9832S	Cell Signaling Technology, Inc., Danvers, USA
HK2	Primary antibody	Rabbit	1:1000	2867S	Cell Signaling Technology, Inc., Danvers, USA
HIF1A	Primary antibody	Rabbit	1:1000	14179S	Cell Signaling Technology, Inc., Danvers, USA
HMGCL	Primary antibody	Rabbit	1:3000	PA5-21996	Invitrogen, Carlsbad, USA
LDHA	Primary antibody	Rabbit	1:1000	3582S	Cell Signaling Technology, Inc., Danvers, USA

Protein	Type	Host	Dilution	Product Number	Supplier
PFKL	Primary antibody	Mouse	1:500	sc-393713	Santa Cruz Biotechnology, Dallas, USA
IRDye® 800CW Goat anti-Rabbit IgG	Secondary antibody	Goat	1:10000	926-32211	LI-COR Biosciences, Lincoln, USA
IRDye® 680RD Goat anti-Mouse IgG	Secondary antibody	Goat	1:10000	926-68070	LI-COR Biosciences, Lincoln, US)

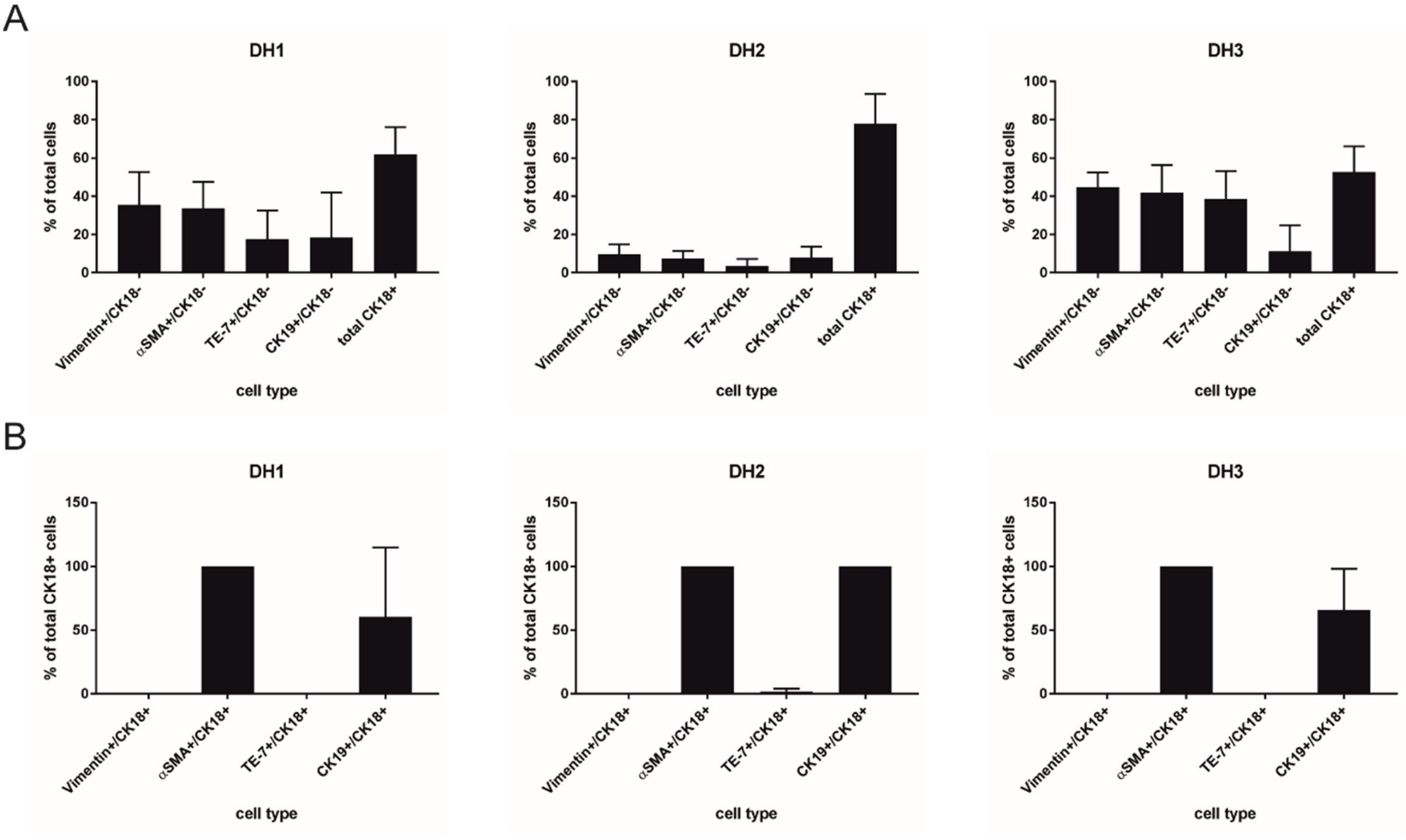

Figure S1. Quantitative evaluation of PHC immunofluorescence staining.

Immunofluorescence staining of PHCs of donor DH1 - DH3 were used for marker quantification. (A) Sole expression of analyzed cell markers for hepatic cells (CK18), fibroblasts (Vimentin, α-SMA, TE-7) and cholangiocytes (CK19). (B) Co-expression of CK18+ with fibroblast (Vimentin, α-SMA, TE-7) and cholangiocyte (CK19) markers. Five random taken images per well were acquired at 20 x magnification using laser scanning microscopy and the cells were counted based on their specific staining. Total cells were counted by Hoechst nuclei staining. Data are shown as means + SD (N = 1, n = 5).

Abbreviations: PHC, primary human hepatoma cell; CK, Cytokeratin; α-SMA, Alpha-smooth muscle actin; TE-7, Anti-fibroblast antibody clone TE-7

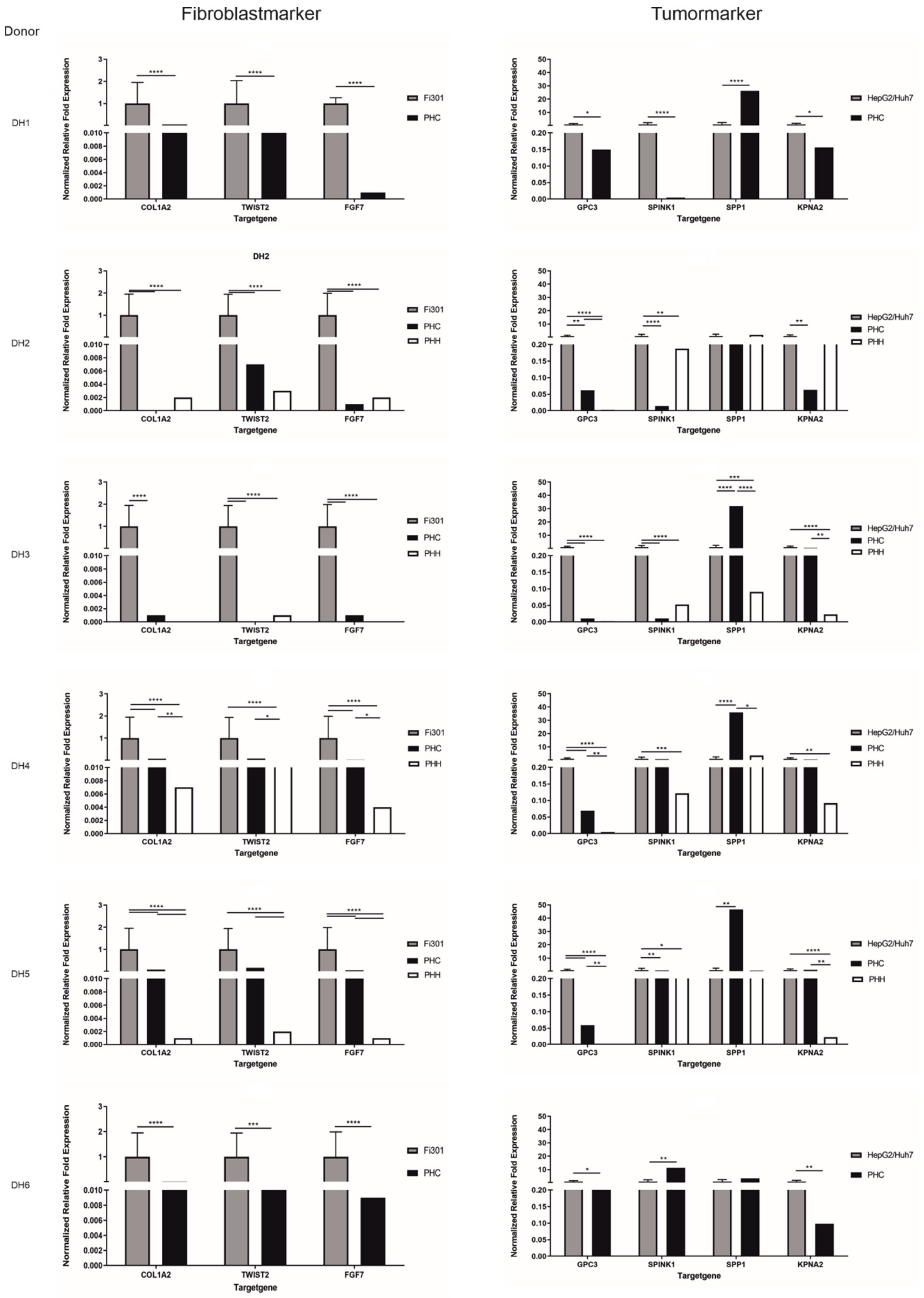

Figure S2. Expression pattern of tumor markers in individual donors.

PHHs (N = 8) and PHCs (N = 6) were analyzed after cell isolation using RT-qPCR. Relative mRNA expression levels of fibroblast markers (*COL1A2*, *TWIST2*, *FGF7*) and tumor markers (*GPC3*,

SPINK1, SPP1, KPNA2) were determined. Fi301 (N = 3) and HepG2/Huh7 (N = 6) cells were used as positive controls. Values are means + SD, n = 3, two-way ANOVA and post hoc Šidák correction or Tukey's test, statistical analyses were conducted on ΔCT values, * $p \leq 0.05$, ** $p \leq 0.01$, *** $p \leq 0.001$, **** $p < 0.0001$.Abbreviations: PHC, primary human hepatoma cell; PHH, primary human hepatocyte; *COL1A2*, collagen type I alpha 2 Chain; *TWIST2*, twist Family BHLH transcription factor 2; *FGF7*, fibroblast growth factor 7; *GPC3*, glypican-3; *SPINK1*, serine protease inhibitor Kazal-type 1; *SPP1*, secreted phosphoprotein-1; *KPNA2*, karyopherin subunit alpha 2

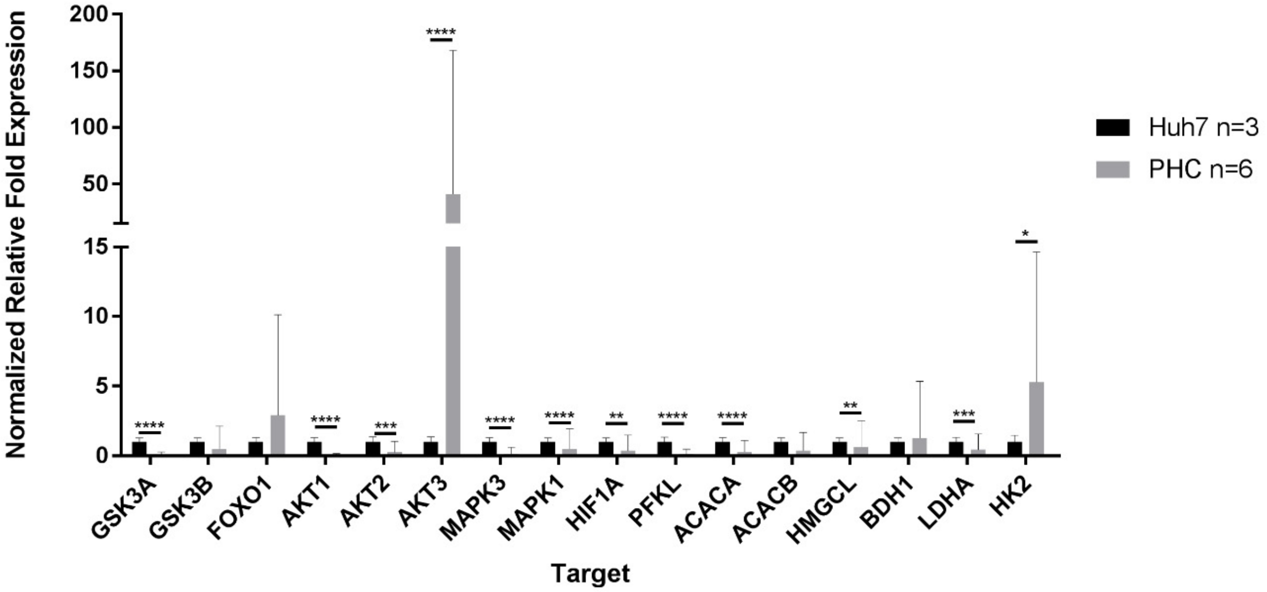

Figure S3. Differentially expressed energy metabolism genes of hepatocellular carcinoma and hepatoma cells.

Cells were cultured for 20 h or snap frozen directly after isolation and relative mRNA expression levels of central actors in hepatocyte metabolism were determined by RT-qPCR. PHCs (N = 6) showed different gene expression patterns in comparison to Huh7 cells (N = 3). Data are shown as means + SD, n = 3, two-way ANOVA and post hoc Šidák correction, statistical analyses were conducted on ΔCT values, * $p \leq 0.05$, ** $p \leq 0.01$, *** $p \leq 0.001$, **** $p < 0.0001$..

Abbreviations: PHH, primary human hepatocyte; PHC, primary human hepatoma cell; HCC, hepatocellular carcinoma; *GSK3A*, glycogen synthase kinase 3 alpha; *GSK3B*, glycogen synthase kinase 3 beta; *FOXO1*, forkhead box O1; *AKT1*, AKT serine/threonine kinase 1; *AKT2*, AKT serine/threonine kinase 2; *AKT3*, AKT serine/threonine kinase 3; *MAPK3*, mitogen-activated protein kinase 3; *MAPK1*, mitogen-activated protein kinase 1; *HIF1A*, hypoxia inducible factor 1 alpha; *PFKL*, phosphofructokinase liver type; *ACACA*, acetyl-CoA carboxylase alpha; *ACACB*, acetyl-CoA carboxylase beta; *HMGCL*, 3-hydroxymethyl-3-methylglutaryl-CoA lyase; *BDH1*, 3-hydroxybutyrate dehydrogenase 1; *LDHA*, lactate dehydrogenase A; *HK2*, hexokinase 2

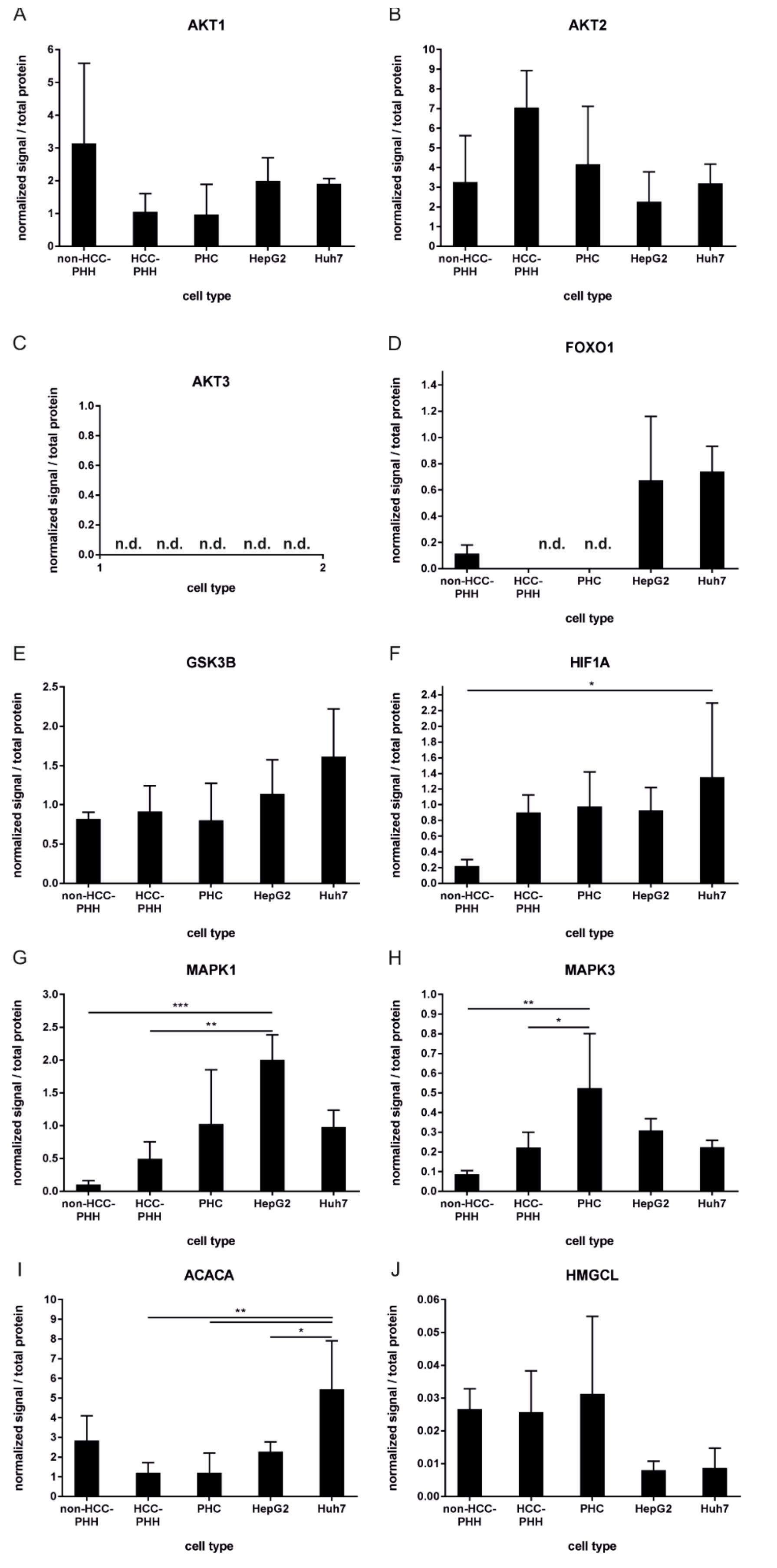

Figure S4. Quantitative evaluation of metabolic target proteins.

Cells were cultured for 20 h or snap frozen directly after isolation and protein expression levels of metabolic targets were determined by Western blotting analysis. (A-J) Healthy hepatocytes (PHH non-HCC, N = 4), PHHs from HCC diagnosed patients (N = 5) and their corresponding PHCs (N = 6) were compared with hepatoma cell lines HepG2 and Huh7 (N = 3) for their characteristic protein expression of different metabolic pathways. Data are shown as means + SD and were normalized on total protein amount, n = 1 - 2, one-way ANOVA and post hoc Tukey's test, * $p \leq 0.05$, ** $p \leq 0.01$, *** $p \leq 0.001$, **** $p < 0.0001$.Abbreviations: n.d., not determined; PHH, primary human hepatocyte; PHC, primary human hepatoma cell; HCC, hepatocellular carcinoma; GSK3B, glycogen synthase kinase 3 beta; FOXO1, forkhead box O1; AKT1, AKT serine/threonine kinase 1; AKT2, AKT serine/threonine kinase 2; AKT3, AKT serine/threonine kinase 3; MAPK3, mitogen-activated protein kinase 3; MAPK1, mitogen-activated protein kinase 1; HIF1A, hypoxia inducible factor 1 alpha; ACACA, acetyl-CoA carboxylase alpha; HMGCL, 3-hydroxymethyl-3-methylglutaryl-CoA lyase

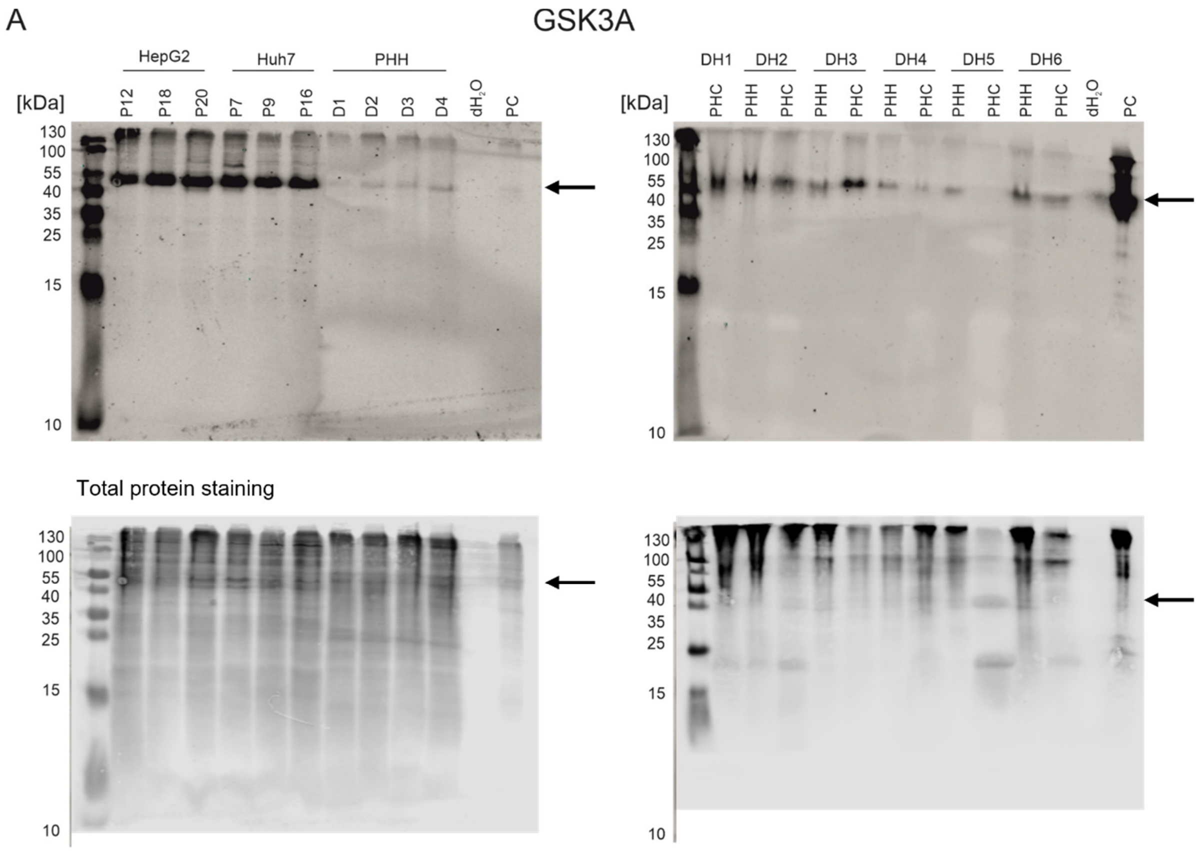

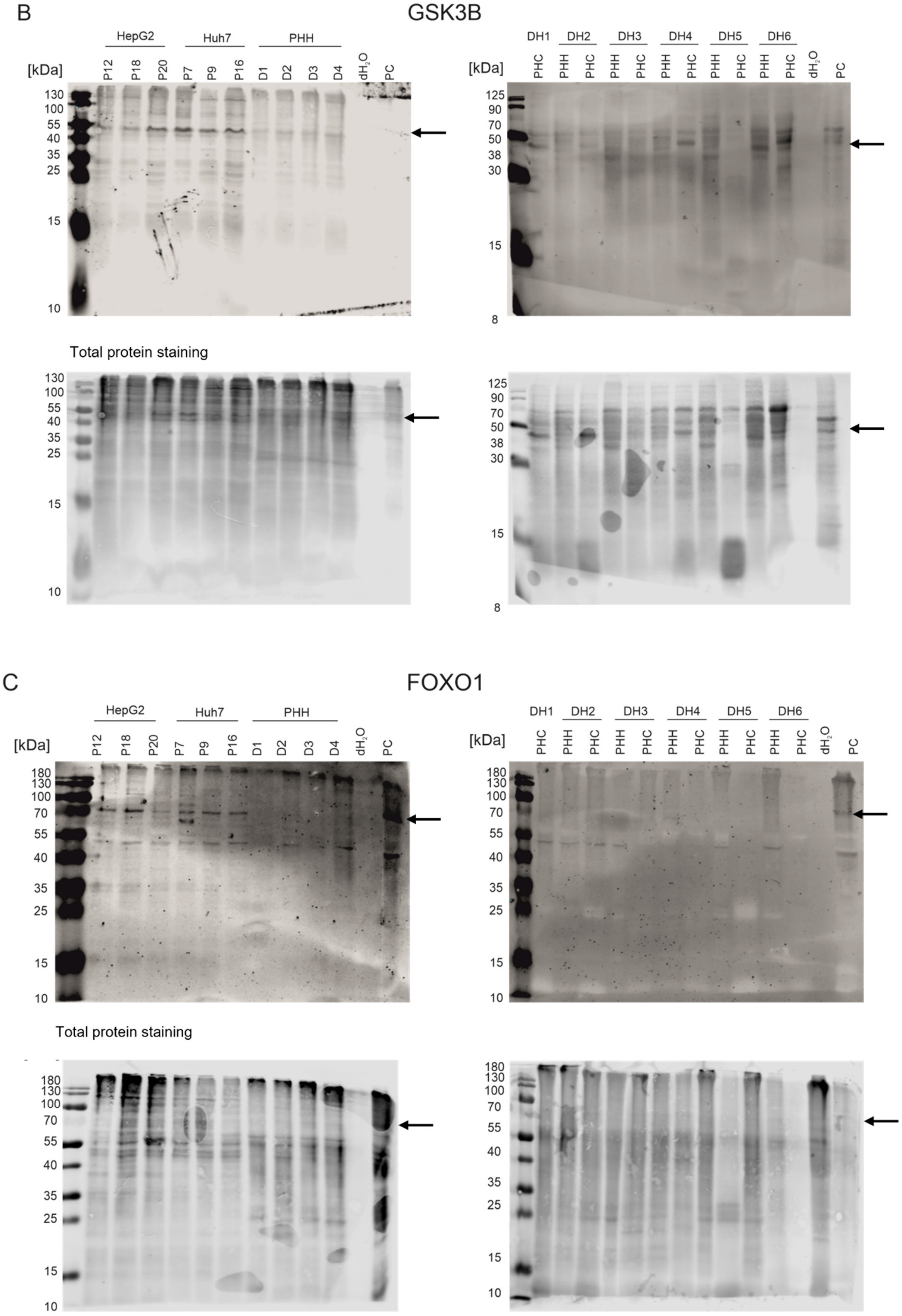
B
GSK3B
HepG2
Huh7
PHH
[kDa]
P12 P18 P20 P7 P9 P16 D1 D2 D3 D4 dH2O PC
130 100 55 40 35 25 15 10
DH1 DH2 DH3 DH4 DH5 DH6
PHC PHH PHC PHH PHC PHH PHC PHH PHC PHH PHC dH2O PC
125 90 70 50 38 30 15 8
Total protein staining
C
FOXO1
180 130 100 70 55 40 35 25 15 10
Total protein staining

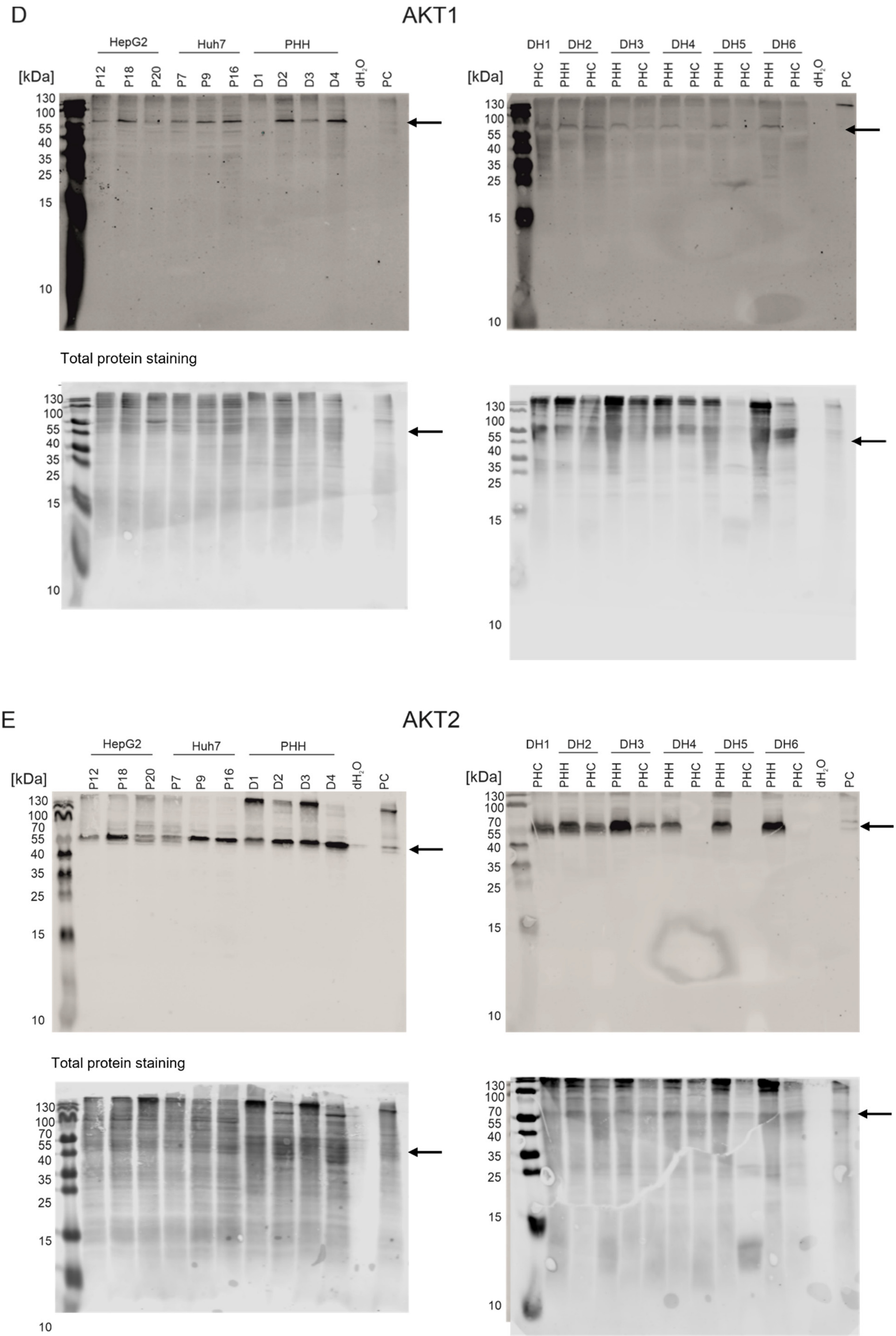
D
AKT1
HepG2
Huh7
PHH
[kDa]
P12
P18
P20
P7
P9
P16
D1
D2
D3
D4
dH2O
PC
DH1
DH2
DH3
DH4
DH5
DH6
PHC
PHH
Total protein staining
130
100
70
55
40
35
25
15
10
E
AKT2

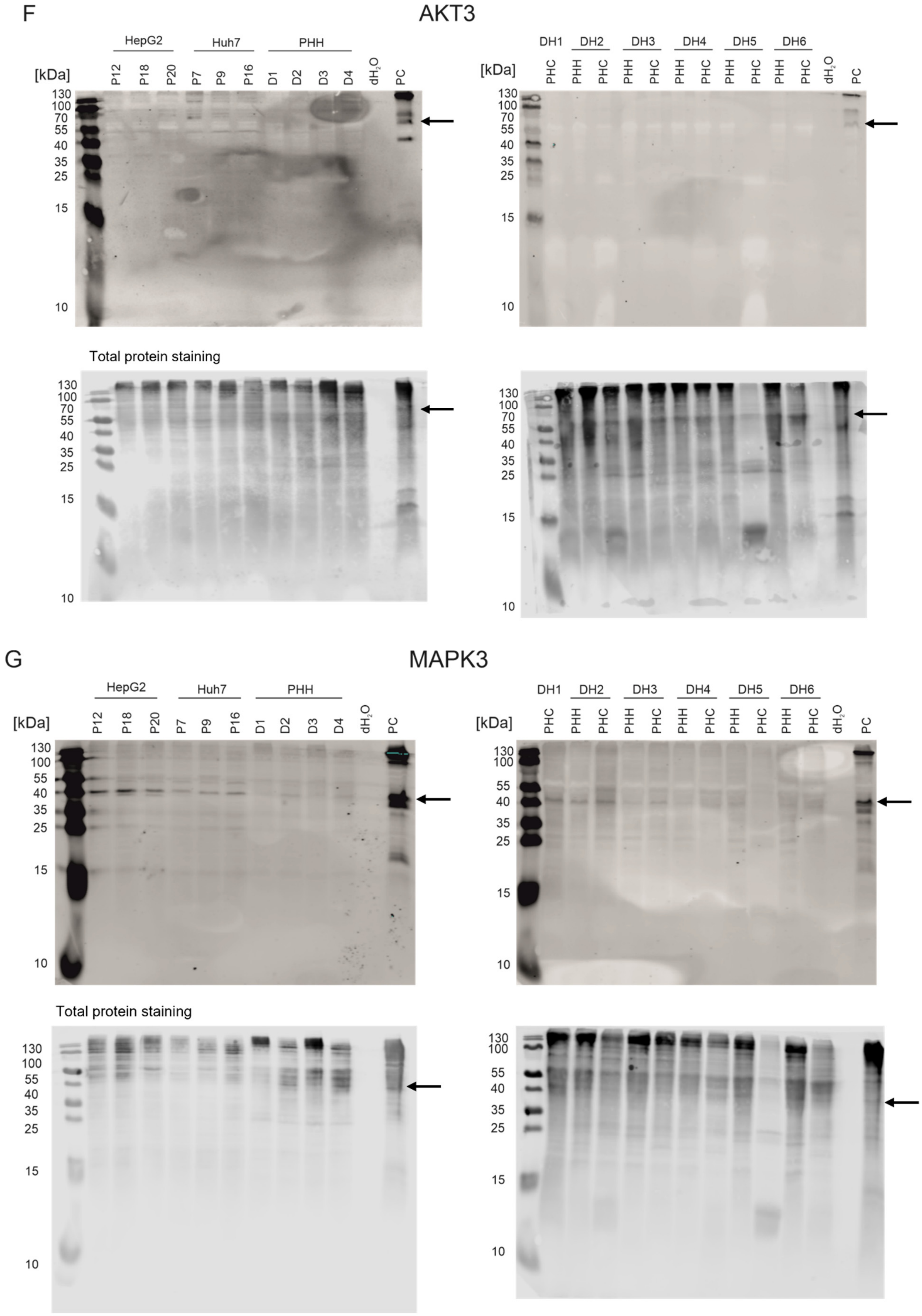
F
AKT3
HepG2
Huh7
PHH
[kDa]
P12 P18 P20 P7 P9 P16 D1 D2 D3 D4 dH$_2$O PC
130 100 70 55 40 35 25 15 10
DH1 DH2 DH3 DH4 DH5 DH6
PHC PHH PHC PHH PHC PHH PHC PHH PHC PHH PHC dH$_2$O PC
Total protein staining
G
MAPK3
HepG2
Huh7
PHH
[kDa]
P12 P18 P20 P7 P9 P16 D1 D2 D3 D4 dH$_2$O PC
130 100 55 40 35 25 15 10
DH1 DH2 DH3 DH4 DH5 DH6
PHC PHH PHC PHH PHC PHH PHC PHH PHC PHH PHC dH$_2$O PC
Total protein staining

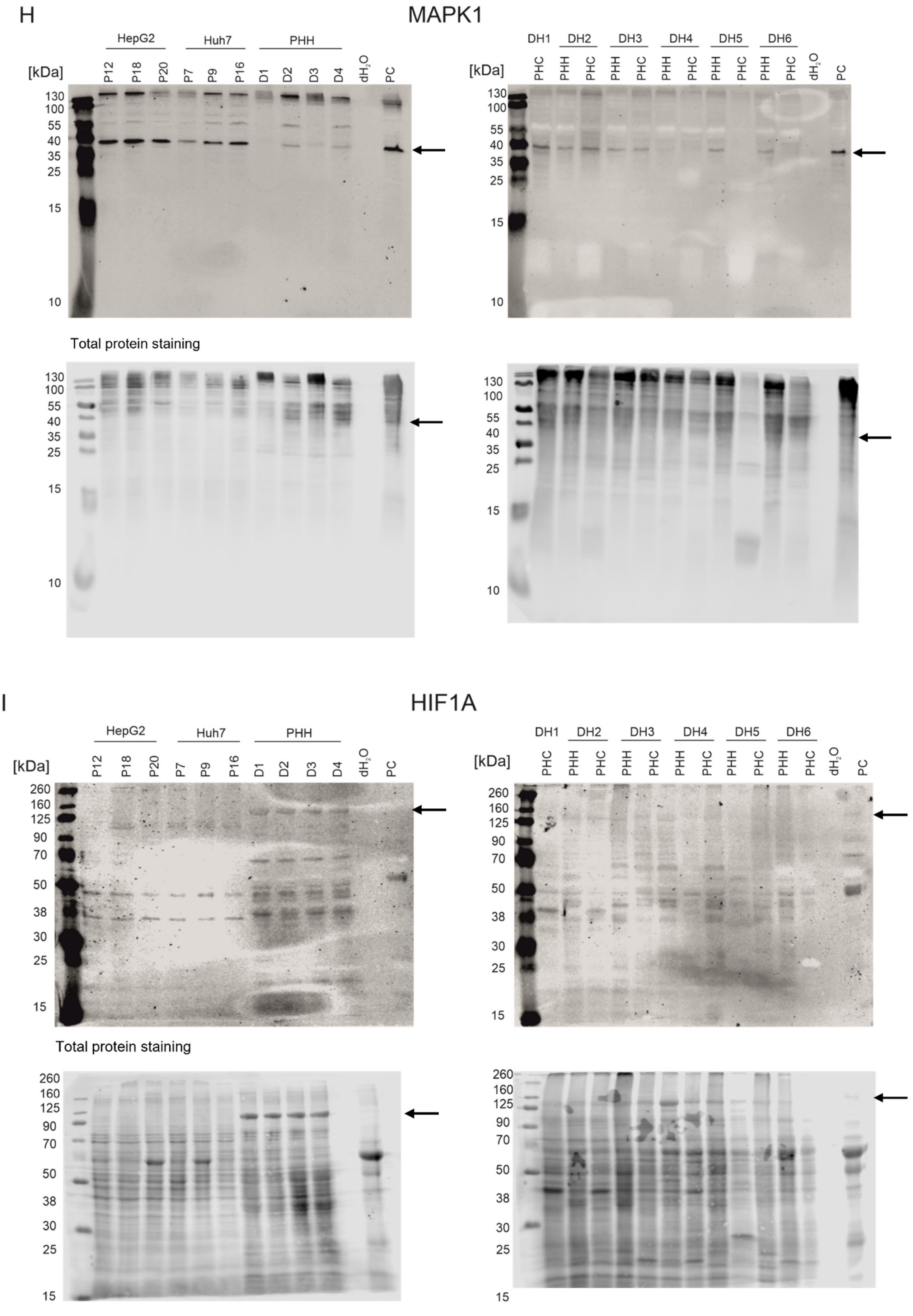
H
MAPK1
HepG2
Huh7
PHH
[kDa]
P12 P18 P20 P7 P9 P16 D1 D2 D3 D4 dH2O PC
130 100 55 40 35 25 15 10
DH1 DH2 DH3 DH4 DH5 DH6
PHC PHH PHC PHH PHC PHH PHC PHH PHC PHH PHC dH2O PC
Total protein staining
I
HIF1A
260 160 125 90 70 50 38 30 25 15

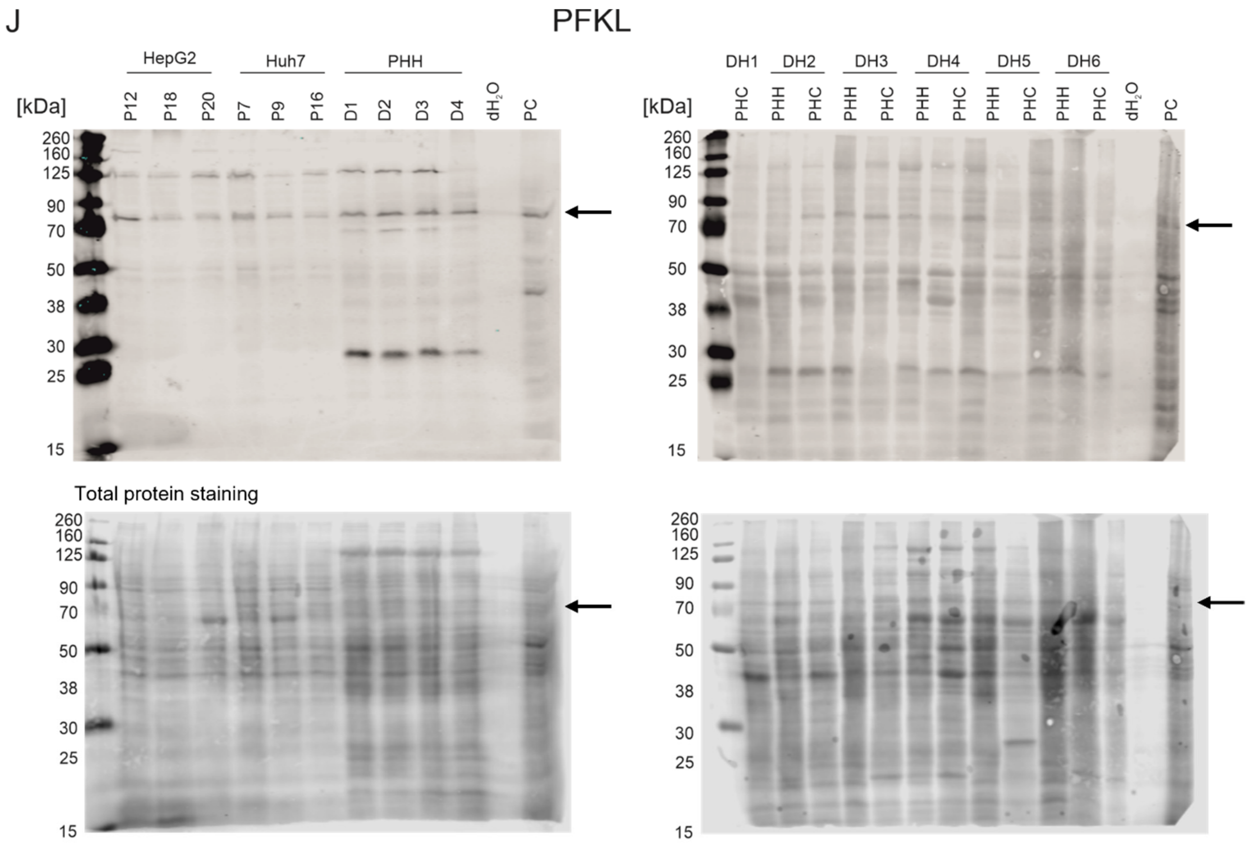
J
PFKL
HepG2
Huh7
PHH
[kDa]
P12
P18
P20
P7
P9
P16
D1
D2
D3
D4
dH$_2$O
PC
260
160
125
90
70
50
38
30
25
15
DH1
DH2
DH3
DH4
DH5
DH6
PHC
PHH
Total protein staining

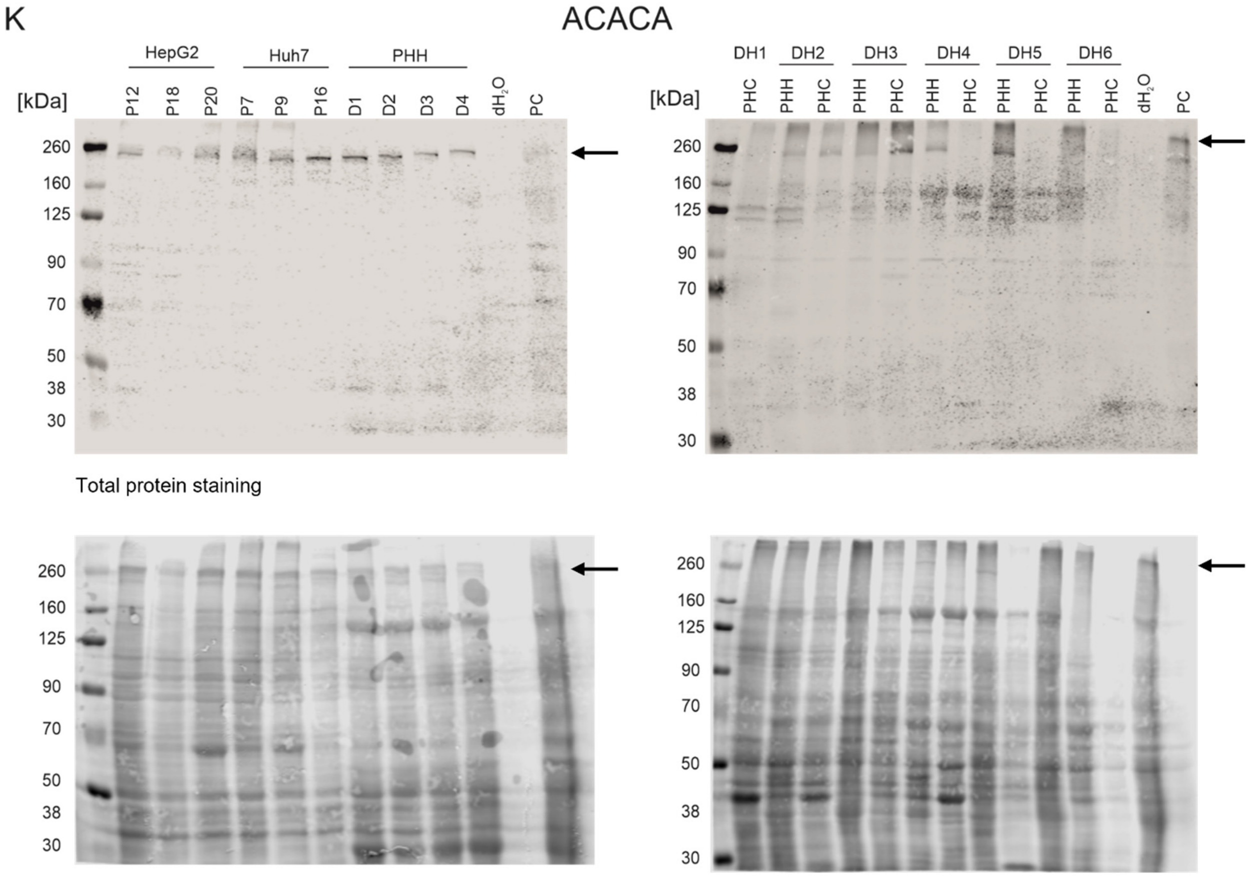
K
ACACA
HepG2
Huh7
PHH
[kDa]
P12
P18
P20
P7
P9
P16
D1
D2
D3
D4
dH$_2$O
PC
260
160
125
90
70
50
38
30
DH1
DH2
DH3
DH4
DH5
DH6
PHC
PHH
Total protein staining

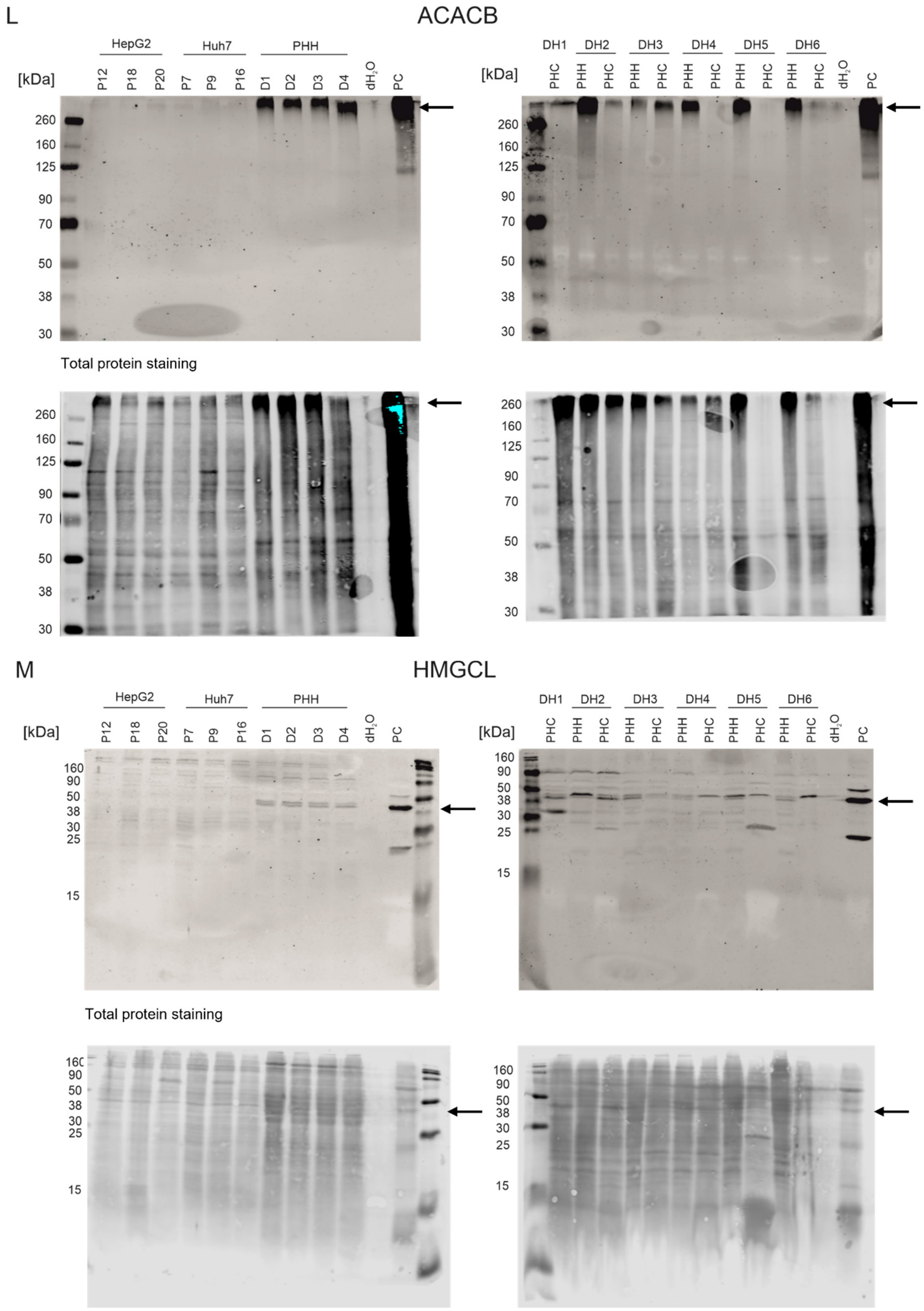
L
ACACB
HepG2
Huh7
PHH
[kDa]
P12 P18 P20 P7 P9 P16 D1 D2 D3 D4 dH2O PC
260 160 125 90 70 50 38 30
DH1 DH2 DH3 DH4 DH5 DH6
PHC PHH PHC PHH PHC PHH PHC PHH PHC PHH PHC dH2O PC
Total protein staining
M
HMGCL
160 90 50 38 30 25 15

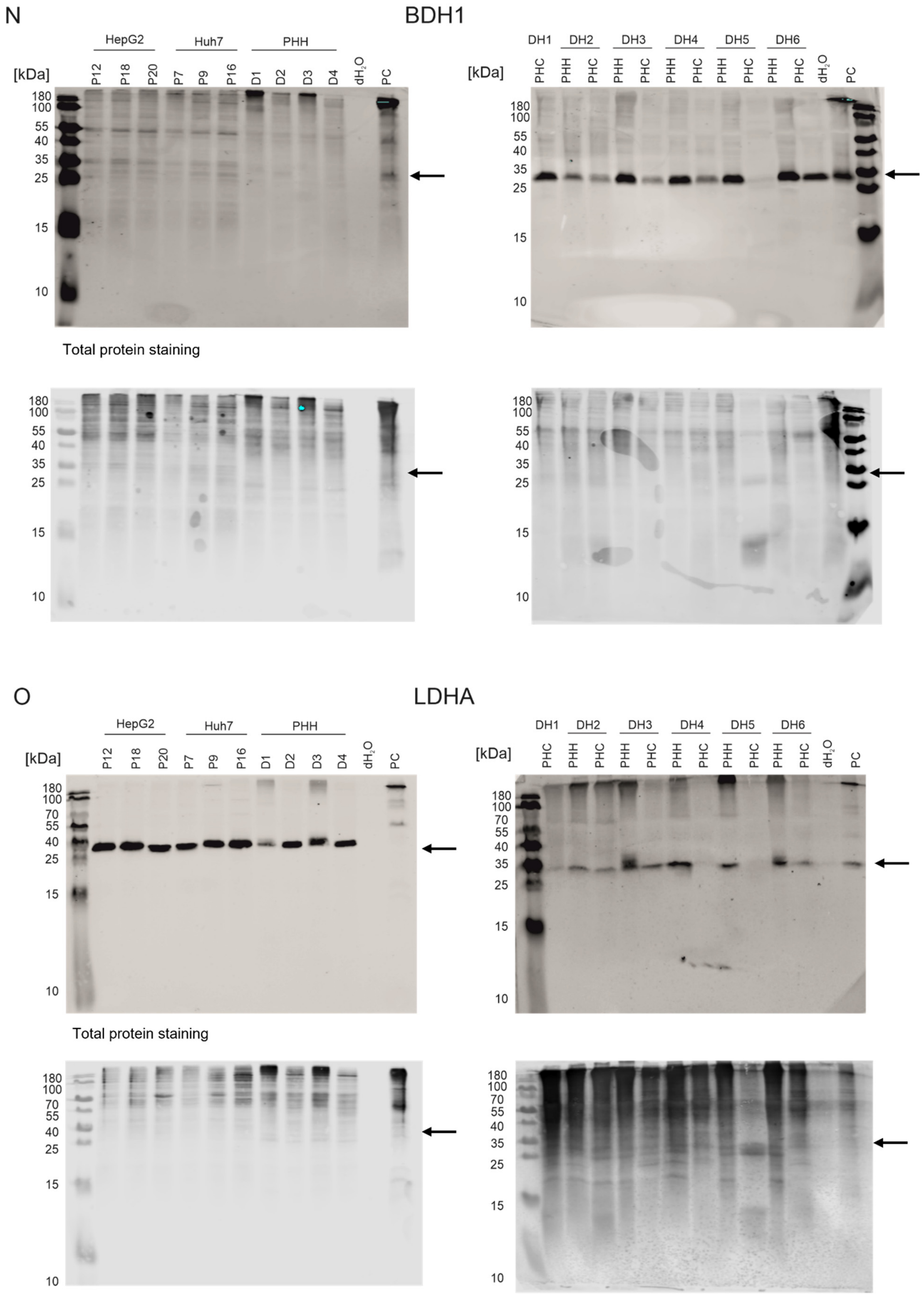
N
BDH1
HepG2
Huh7
PHH
[kDa]
P12 P18 P20 P7 P9 P16 D1 D2 D3 D4 dH2O PC
180 100 55 40 35 25 15 10
DH1 DH2 DH3 DH4 DH5 DH6
PHC PHH PHC PHH PHC PHH PHC PHH PHC PHH PHC dH2O PC
Total protein staining
O
LDHA
180 100 70 55 40 35 25 15 10

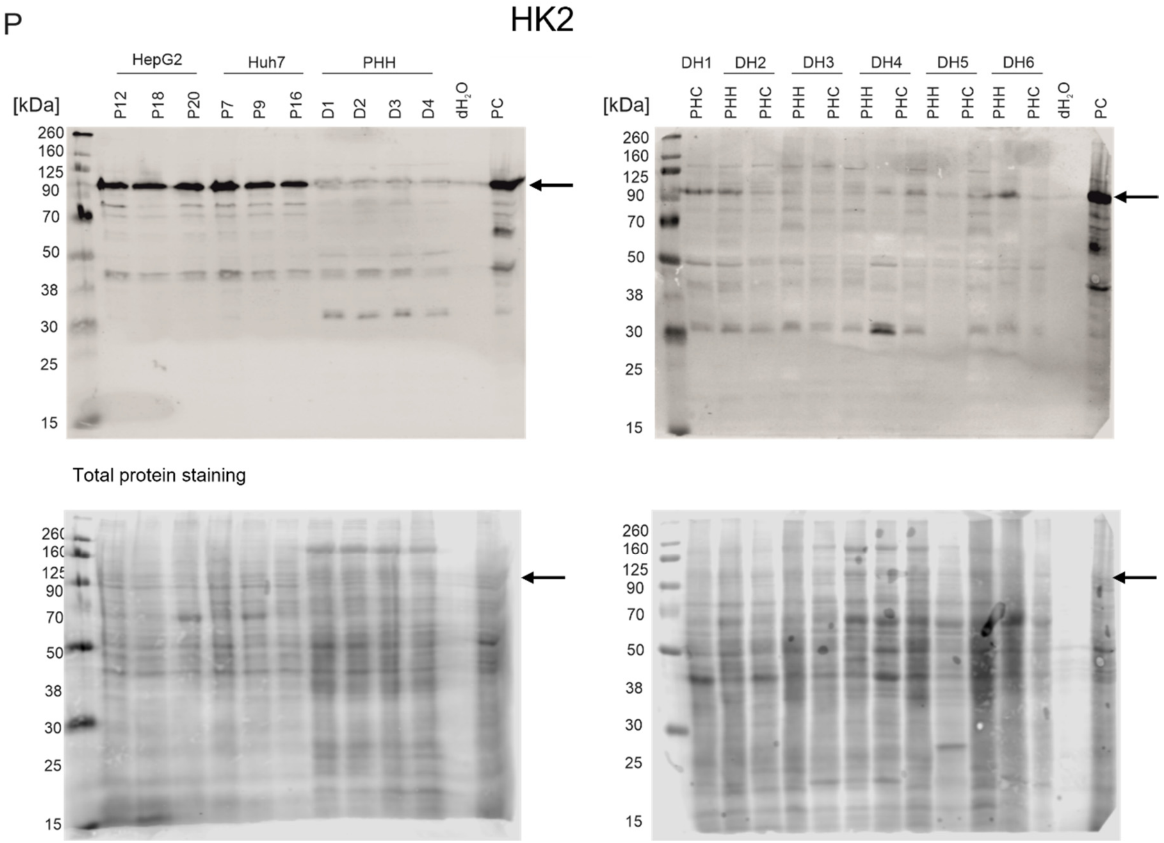

Figure S5. Western Blots of analyzed proteins.

Proteins were separated using SDS-PAGE on gels (8 - 15 %) and transferred to nitrocellulose membranes via tank blot system. Membranes were stained with primary antibodies overnight. For detection, fluorescence coupled secondary antibodies were used and incubated for 1 h. Membranes were dried and proteins were quantified using Odyssey 9120 Imaging System.

Abbreviations: PHH, primary human hepatocyte; PHC, primary human hepatoma cell; HCC, hepatocellular carcinoma; GSK3A, glycogen synthase kinase 3 alpha; GSK3B, glycogen synthase kinase 3 beta; FOXO1, forkhead box O1; AKT1, AKT serine/threonine kinase 1; AKT2, AKT serine/threonine kinase 2; AKT3, AKT serine/threonine kinase 3; MAPK3, mitogen-activated protein kinase 3; MAPK1, mitogen-activated protein kinase 1; HIF1A, hypoxia inducible factor 1 alpha; PFKL, phosphofructokinase liver type; ACACA, acetyl-CoA carboxylase alpha; ACACB, acetyl-CoA carboxylase beta; HMGCL, 3-hydroxymethyl-3-methylglutaryl-CoA lyase; BDH1, 3-hydroxybutyrate dehydrogenase 1; LDHA, lactate dehydrogenase A; HK2, hexokinase 2

Table S5. Values of test statistics of fibroblast marker (Figure 2A). P values of 0.05 or less were considered significant (*** $p \leq 0.001$, **** $p < 0.0001$).

**Number of families**	3							
**Number of comparisons per family**	3							
**Alpha**	0,05							
**Tukey's multiple comparisons test**	**Predicted (LS) mean diff,**	**95,00% CI of diff,**	**Below threshold?**	**Summary**	**Adjusted P Value**			
Col1A2								
Fi301 n=3 vs. PHC n=6	-5,824	-7,752 to -3,897	Yes	****	<0,0001			
Fi301 n=3 vs. PHH n=8	-10,51	-12,53 to -8,499	Yes	****	<0,0001			
PHC n=6 vs. PHH n=8	-4,69	-6,461 to -2,920	Yes	****	<0,0001			
TWIST2								
Fi301 n=3 vs. PHC n=6	-4,114	-5,980 to -2,248	Yes	****	<0,0001			
Fi301 n=3 vs. PHH n=8	-6,478	-8,265 to -4,691	Yes	****	<0,0001			
PHC n=6 vs. PHH n=8	-2,364	-3,789 to -0,9381	Yes	***	0,0004			
FGF7								
Fi301 n=3 vs. PHC n=6	-7,856	-9,722 to -5,989	Yes	****	<0,0001			
Fi301 n=3 vs. PHH n=8	-10,24	-12,03 to -8,457	Yes	****	<0,0001			
PHC n=6 vs. PHH n=8	-2,389	-3,814 to -0,9632	Yes	***	0,0003			
**Test details**	**Predicted (LS) mean 1**	**Predicted (LS) mean 2**	**Predicted (LS) mean diff,**	**SE of diff,**	**N1**	**N2**	**q**	**DF**
Col1A2								
Fi301 n=3 vs. PHC n=6	3,249	9,073	-5,824	0,813	9	15	10,13	129
Fi301 n=3 vs. PHH n=8	3,249	13,76	-10,51	0,8502	9	12	17,49	129
PHC n=6 vs. PHH n=8	9,073	13,76	-4,69	0,7468	15	12	8,882	129
TWIST2								
Fi301 n=3 vs. PHC n=6	6,364	10,48	-4,114	0,7872	9	18	7,391	129
Fi301 n=3 vs. PHH n=8	6,364	12,84	-6,478	0,7537	9	24	12,15	129
PHC n=6 vs. PHH n=8	10,48	12,84	-2,364	0,6012	18	24	5,56	129
FGF7								
Fi301 n=3 vs. PHC n=6	2,873	10,73	-7,856	0,7872	9	18	14,11	129
Fi301 n=3 vs. PHH n=8	2,873	13,12	-10,24	0,7537	9	24	19,22	129
PHC n=6 vs. PHH n=8	10,73	13,12	-2,389	0,6012	18	24	5,619	129

Table S6. Values of test statistics of tumor marker (Figure 2B). P values of 0.05 or less were considered significant (* $p \leq 0.05$, ** $p \leq 0.01$, **** $p < 0.0001$).

**Number of families**	4							
**Number of comparisons per family**	3							
**Alpha**	0,05							
**Tukey's multiple comparisons test**	**Predicted (LS) mean diff,**	**95,00% CI of diff,**	**Below threshold?**	**Summary**	**Adjusted P Value**			
GPC3								
HepG2/Huh7 n=6 vs. PHC n=6	-4,514	-6,050 to -2,978	Yes	****	<0,0001			
HepG2/Huh7 n=6 vs. PHH n=8	-9,165	-10,60 to -7,728	Yes	****	<0,0001			
PHC n=6 vs. PHH n=8	-4,651	-6,088 to -3,214	Yes	****	<0,0001			
SPINK1								
HepG2/Huh7 n=6 vs. PHC n=6	-3,737	-5,273 to -2,201	Yes	****	<0,0001			
HepG2/Huh7 n=6 vs. PHH n=8	-2,203	-3,640 to -0,7660	Yes	**	0,0011			
PHC n=6 vs. PHH n=8	1,534	0,09686 to 2,971	Yes	*	0,0333			
SPP1								
HepG2/Huh7 n=6 vs. PHC n=6	3,07	1,534 to 4,606	Yes	****	<0,0001			
HepG2/Huh7 n=6 vs. PHH n=8	-1,213	-2,650 to 0,2237	No	ns	0,1165			
PHC n=6 vs. PHH n=8	-4,283	-5,720 to -2,846	Yes	****	<0,0001			
KPNA2								
HepG2/Huh7 n=6 vs. PHC n=6	-1,955	-3,491 to -0,4191	Yes	**	0,0083			
HepG2/Huh7 n=6 vs. PHH n=8	-3,976	-5,413 to -2,540	Yes	****	<0,0001			
PHC n=6 vs. PHH n=8	-2,021	-3,458 to -0,5843	Yes	**	0,003			
**Test details**	**Predicted (LS) mean 1**	**Predicted (LS) mean 2**	**Predicted (LS) mean diff,**	**SE of diff,**	**N1**	**N2**	**q**	**DF**
GPC3								
HepG2/Huh7 n=6 vs. PHC n=6	-1,084	3,43	-4,514	0,6511	18	18	9,804	228
HepG2/Huh7 n=6 vs. PHH n=8	-1,084	8,081	-9,165	0,6091	18	24	21,28	228
PHC n=6 vs. PHH n=8	3,43	8,081	-4,651	0,6091	18	24	10,8	228
SPINK1								
HepG2/Huh7 n=6 vs. PHC n=6	4,265	8,002	-3,737	0,6511	18	18	8,116	228
HepG2/Huh7 n=6 vs. PHH n=8	4,265	6,468	-2,203	0,6091	18	24	5,115	228
PHC n=6 vs. PHH n=8	8,002	6,468	1,534	0,6091	18	24	3,561	228
SPP1								
HepG2/Huh7 n=6 vs. PHC n=6	4,014	0,9434	3,07	0,6511	18	18	6,668	228
HepG2/Huh7 n=6 vs. PHH n=8	4,014	5,227	-1,213	0,6091	18	24	2,817	228
PHC n=6 vs. PHH n=8	0,9434	5,227	-4,283	0,6091	18	24	9,945	228
KPNA2								
HepG2/Huh7 n=6 vs. PHC n=6	4,436	6,391	-1,955	0,6511	18	18	4,247	228
HepG2/Huh7 n=6 vs. PHH n=8	4,436	8,412	-3,976	0,6091	18	24	9,233	228
PHC n=6 vs. PHH n=8	6,391	8,412	-2,021	0,6091	18	24	4,693	228

Table S7. Values of test statistics of RT-qPCR analyses of non-HCC-PHHs and HCC-PHHs (Figure 3A). P values of 0.05 or less were considered significant (* $p \leq 0.05$, ** $p \leq 0.01$, *** $p \leq 0.001$, **** $p < 0.0001$).

**Number of families**	1							
**Number of comparisons per family**	16							
**Alpha**	0,05							
**Sidak's multiple comparisons test**	**Mean Diff,**	**95,00% CI of diff,**	**Significant?**	**Summary**	**Adjusted P Value**			
PHH healthy n=4 - PHH HCC n=9								
GSK3A	-1,987	-3,222 to -0,7516	Yes	****	<0,0001			
GSK3B	0,7716	-0,4565 to 2	No	ns	0,6495			
FOXO1	3,498	2,27 to 4,726	Yes	****	<0,0001			
AKT1	-2,987	-4,253 to -1,721	Yes	****	<0,0001			
AKT2	-1,401	-2,629 to -0,1731	Yes	*	0,0124			
AKT3	1,178	-0,05688 to 2,414	No	ns	0,0757			
MAPK3	-1,878	-3,106 to -0,6502	Yes	***	0,0001			
MAPK1	-0,8239	-2,052 to 0,4042	No	ns	0,5412			
HIF1A	1,403	0,1751 to 2,631	Yes	*	0,0122			
PFKL	-1,619	-2,848 to -0,3913	Yes	**	0,0017			
ACACA	-0,529	-1,78 to 0,7225	No	ns	0,9776			
ACACB	-0,9131	-2,141 to 0,3149	No	ns	0,3666			
HMGCL	-1,377	-2,605 to -0,1485	Yes	*	0,0153			
BDH1	2,294	1,066 to 3,522	Yes	****	<0,0001			
LDHA	-0,4486	-1,677 to 0,7795	No	ns	0,9948			
HK2	7,024	5,796 to 8,252	Yes	****	<0,0001			
**Test details**	**Mean 1**	**Mean 2**	**Mean Diff,**	**SE of diff,**	**N1**	**N2**	**t**	**DF**
PHH healthy n=4 - PHH HCC n=9								
GSK3A	7,382	9,369	-1,987	0,4173	12	26	4,761	586
GSK3B	8,824	8,052	0,7716	0,4149	12	27	1,86	586
FOXO1	9,051	5,553	3,498	0,4149	12	27	8,431	586
AKT1	5,048	8,035	-2,987	0,4277	11	27	6,983	586
AKT2	7,294	8,695	-1,401	0,4149	12	27	3,377	586
AKT3	13,17	11,99	1,178	0,4173	12	26	2,824	586
MAPK3	8,891	10,77	-1,878	0,4149	12	27	4,527	586
MAPK1	7,302	8,125	-0,8239	0,4149	12	27	1,986	586
HIF1A	8,099	6,695	1,403	0,4149	12	27	3,382	586
PFKL	8,172	9,791	-1,619	0,4149	12	27	3,903	586
ACACA	10,55	11,08	-0,529	0,4228	12	24	1,251	586
ACACB	7,757	8,67	-0,9131	0,4149	12	27	2,201	586
HMGCL	5,538	6,914	-1,377	0,4149	12	27	3,318	586
BDH1	9,571	7,277	2,294	0,4149	12	27	5,53	586
LDHA	2,911	3,36	-0,4486	0,4149	12	27	1,081	586
HK2	17,69	10,67	7,024	0,4149	12	27	16,93	586

Table S8. Values of test statistics of RT-qPCR analyses of HCC-PHHs and HepG2 cells (Figure 3B). P values of 0.05 or less were considered significant (* $p \leq 0.05$, ** $p \leq 0.01$, **** $p < 0.0001$).

**Number of families**	1							
**Number of comparisons per family**	16							
**Alpha**	0,05							
**Sidak's multiple comparisons test**	**Mean Diff,**	**95,00% CI of diff,**	**Significant?**	**Summary**	**Adjusted P Value**			
PHH HCC n=9 - HepG2 n=3								
GSK3A	4,736	3,311 to 6,16	Yes	****	<0,0001			
GSK3B	2,225	0,8073 to 3,643	Yes	****	<0,0001			
FOXO1	-1,1	-2,518 to 0,3177	No	ns	0,2991			
AKT1	4,659	3,241 to 6,077	Yes	****	<0,0001			
AKT2	2,614	1,197 to 4,032	Yes	****	<0,0001			
AKT3	-1,584	-3,009 to -0,1598	Yes	*	0,0168			
MAPK3	5,757	4,339 to 7,175	Yes	****	<0,0001			
MAPK1	3,258	1,841 to 4,676	Yes	****	<0,0001			
HIF1A	2,724	1,306 to 4,141	Yes	****	<0,0001			
PFKL	4,806	3,388 to 6,224	Yes	****	<0,0001			
ACACA	4,055	2,615 to 5,495	Yes	****	<0,0001			
ACACB	0,3426	-1,075 to 1,76	No	ns	>0,9999			
HMGCL	0,6741	-0,7438 to 2,092	No	ns	0,9383			
BDH1	-0,4025	-1,82 to 1,015	No	ns	0,9997			
LDHA	1,857	0,4392 to 3,275	Yes	**	0,0019			
HK2	-1,222	-2,64 to 0,1959	No	ns	0,162			
**Test details**	**Mean 1**	**Mean 2**	**Mean Diff,**	**SE of diff,**	**N1**	**N2**	**t**	**DF**
PHH HCC n=9 - HepG2 n=3								
GSK3A	9,369	4,633	4,736	0,4811	26	9	9,843	539
GSK3B	8,052	5,827	2,225	0,4788	27	9	4,647	539
FOXO1	5,553	6,653	-1,1	0,4788	27	9	2,298	539
AKT1	8,035	3,376	4,659	0,4788	27	9	9,73	539
AKT2	8,695	6,081	2,614	0,4788	27	9	5,46	539
AKT3	11,99	13,58	-1,584	0,4811	26	9	3,293	539
MAPK3	10,77	5,013	5,757	0,4788	27	9	12,02	539
MAPK1	8,125	4,867	3,258	0,4788	27	9	6,805	539
HIF1A	6,695	3,972	2,724	0,4788	27	9	5,688	539
PFKL	9,791	4,985	4,806	0,4788	27	9	10,04	539
ACACA	11,08	7,023	4,055	0,4862	24	9	8,339	539
ACACB	8,67	8,327	0,3426	0,4788	27	9	0,7156	539
HMGCL	6,914	6,24	0,6741	0,4788	27	9	1,408	539
BDH1	7,277	7,68	-0,4025	0,4788	27	9	0,8407	539
LDHA	3,36	1,503	1,857	0,4788	27	9	3,878	539
HK2	10,67	11,89	-1,222	0,4788	27	9	2,552	539

Table S9. Values of test statistics of RT-qPCR analyses of HCC-PHHs and Huh7 cells (Figure 3B). P values of 0.05 or less were considered significant (*** $p \leq 0.001$, **** $p < 0.0001$).

**Number of families**	1							
**Number of comparisons per family**	16							
**Alpha**	0,05							
**Sidak's multiple comparisons test**	**Mean Diff,**	**95,00% CI of diff,**	**Significant?**	**Summary**	**Adjusted P Value**			
PHH HCC n=9 - Huh7 n=3								
GSK3A	4,723	3,313 to 6,133	Yes	****	<0,0001			
GSK3B	2,181	0,7779 to 3,584	Yes	****	<0,0001			
FOXO1	-1,108	-2,512 to 0,2949	No	ns	0,2727			
AKT1	4,653	3,25 to 6,056	Yes	****	<0,0001			
AKT2	2,734	1,331 to 4,137	Yes	****	<0,0001			
AKT3	-2,8	-4,274 to -1,326	Yes	****	<0,0001			
MAPK3	5,384	3,981 to 6,788	Yes	****	<0,0001			
MAPK1	3,169	1,766 to 4,573	Yes	****	<0,0001			
HIF1A	2,816	1,413 to 4,22	Yes	****	<0,0001			
PFKL	4,737	3,334 to 6,14	Yes	****	<0,0001			
ACACA	4,087	2,662 to 5,512	Yes	****	<0,0001			
ACACB	0,5185	-0,8848 to 1,922	No	ns	0,9941			
HMGCL	0,6188	-0,7845 to 2,022	No	ns	0,9671			
BDH1	-0,5014	-1,905 to 0,9018	No	ns	0,9959			
LDHA	2,088	0,6844 to 3,491	Yes	***	0,0002			
HK2	1,321	-0,08227 to 2,724	No	ns	0,0845			
**Test details**	**Mean 1**	**Mean 2**	**Mean Diff,**	**SE of diff,**	**N1**	**N2**	**t**	**DF**
PHH HCC n=9 - Huh7 n=3								
GSK3A	9,369	4,646	4,723	0,4762	26	9	9,918	538
GSK3B	8,052	5,871	2,181	0,4739	27	9	4,603	538
FOXO1	5,553	6,661	-1,108	0,4739	27	9	2,339	538
AKT1	8,035	3,382	4,653	0,4739	27	9	9,818	538
AKT2	8,695	5,961	2,734	0,4739	27	9	5,769	538
AKT3	11,99	14,79	-2,8	0,4978	26	8	5,624	538
MAPK3	10,77	5,385	5,384	0,4739	27	9	11,36	538
MAPK1	8,125	4,956	3,169	0,4739	27	9	6,688	538
HIF1A	6,695	3,879	2,816	0,4739	27	9	5,943	538
PFKL	9,791	5,054	4,737	0,4739	27	9	9,996	538
ACACA	11,08	6,991	4,087	0,4812	24	9	8,492	538
ACACB	8,67	8,151	0,5185	0,4739	27	9	1,094	538
HMGCL	6,914	6,295	0,6188	0,4739	27	9	1,306	538
BDH1	7,277	7,779	-0,5014	0,4739	27	9	1,058	538
LDHA	3,36	1,272	2,088	0,4739	27	9	4,405	538
HK2	10,67	9,346	1,321	0,4739	27	9	2,788	538

Table S10. Values of test statistics of RT-qPCR analyses of HCC-PHHs and PHCs (Figure 3C). P values of 0.05 or less were considered significant (** $p \leq 0.01$, *** $p \leq 0.001$, **** $p < 0.0001$).

**Number of families**	1							
**Number of comparisons per family**	16							
**Alpha**	0,05							
**Sidak's multiple comparisons test**	**Predicted (LS) mean diff,**	**95,00% CI of diff,**	**Significant?**	**Summary**	**Adjusted P Value**			
PHH HCC n=9 - PHC n=6								
GSK3A	0,8328	-0,4177 to 2,083	No	ns	0,5541			
GSK3B	0,9777	-0,2423 to 2,198	No	ns	0,2525			
FOXO1	-0,5208	-1,741 to 0,6992	No	ns	0,9756			
AKT1	0,0286	-1,191 to 1,249	No	ns	>0,9999			
AKT2	0,8477	-0,3936 to 2,089	No	ns	0,5112			
AKT3	2,173	0,8729 to 3,473	Yes	****	<0,0001			
MAPK3	1,656	0,4146 to 2,897	Yes	**	0,0014			
MAPK1	1,051	-0,1693 to 2,271	No	ns	0,163			
HIF1A	1,137	-0,08346 to 2,357	No	ns	0,0919			
PFKL	0,7362	-0,4838 to 1,956	No	ns	0,7112			
ACACA	1,91	0,6392 to 3,181	Yes	***	0,0002			
ACACB	-0,728	-1,993 to 0,5369	No	ns	0,7753			
HMGCL	-1,107	-2,327 to 0,1135	No	ns	0,1131			
BDH1	-0,7901	-2,010 to 0,4299	No	ns	0,601			
LDHA	0,1354	-1,085 to 1,355	No	ns	>0,9999			
HK2	2,766	1,546 to 3,986	Yes	****	<0,0001			
**Test details**	**Predicted (LS) mean 1**	**Predicted (LS) mean 2**	**ted (LS) mea**	**SE of diff,**	**N1**	**N2**	**t**	**DF**
PHH HCC n=9 - PHC n=6								
GSK3A	9,369	8,536	0,8328	0,4227	26	17	1,97	674
GSK3B	8,052	7,074	0,9777	0,4124	27	18	2,371	674
FOXO1	5,553	6,074	-0,5208	0,4124	27	18	1,263	674
AKT1	8,035	8,006	0,0286	0,4124	27	18	0,06935	674
AKT2	8,695	7,848	0,8477	0,4196	27	17	2,02	674
AKT3	11,99	9,819	2,173	0,4394	26	15	4,945	674
MAPK3	10,77	9,114	1,656	0,4196	27	17	3,947	674
MAPK1	8,125	7,075	1,051	0,4124	27	18	2,548	674
HIF1A	6,695	5,559	1,137	0,4124	27	18	2,756	674
PFKL	9,791	9,055	0,7362	0,4124	27	18	1,785	674
ACACA	11,08	9,168	1,91	0,4296	24	17	4,446	674
ACACB	8,67	9,398	-0,728	0,4276	27	16	1,703	674
HMGCL	6,914	8,021	-1,107	0,4124	27	18	2,683	674
BDH1	7,277	8,067	-0,7901	0,4124	27	18	1,916	674
LDHA	3,36	3,225	0,1354	0,4124	27	18	0,3283	674
HK2	10,67	7,901	2,766	0,4124	27	18	6,707	674

Table S11. Values of test statistics of RT-qPCR analyses of HepG2 cells and PHCs (Figure 3D). P values of 0.05 or less were considered significant (** $p \leq 0.01$, **** $p < 0.0001$).

**Number of families**	1							
**Number of comparisons per family**	16							
**Alpha**	0,05							
**Sidak's multiple comparisons test**	**Mean Diff,**	**95,00% CI of diff,**	**Significant?**	**Summary**	**Adjusted P Value**			
HepG2 n=3 - PHC n=6								
GSK3A	-3,903	-5,227 to -2,579	Yes	****	<0,0001			
GSK3B	-1,247	-2,558 to 0,06365	No	ns	0,0773			
FOXO1	0,5794	-0,7316 to 1,89	No	ns	0,9661			
AKT1	-4,63	-5,941 to -3,319	Yes	****	<0,0001			
AKT2	-1,767	-3,091 to -0,4429	Yes	**	0,0014			
AKT3	3,757	2,403 to 5,111	Yes	****	<0,0001			
MAPK3	-4,101	-5,425 to -2,777	Yes	****	<0,0001			
MAPK1	-2,208	-3,519 to -0,8967	Yes	****	<0,0001			
HIF1A	-1,587	-2,898 to -0,2761	Yes	**	0,0059			
PFKL	-4,07	-5,381 to -2,759	Yes	****	<0,0001			
ACACA	-2,145	-3,469 to -0,8211	Yes	****	<0,0001			
ACACB	-1,071	-2,409 to 0,2674	No	ns	0,2536			
HMGCL	-1,781	-3,092 to -0,4696	Yes	**	0,0011			
BDH1	-0,3875	-1,699 to 0,9235	No	ns	0,9995			
LDHA	-1,722	-3,033 to -0,4106	Yes	**	0,0018			
HK2	3,988	2,677 to 5,299	Yes	****	<0,0001			
**Test details**	**Mean 1**	**Mean 2**	**Mean Diff,**	**SE of diff,**	**N1**	**N2**	**t**	**DF**
HepG2 n=3 - PHC n=6								
GSK3A	4,633	8,536	-3,903	0,4463	9	17	8,745	391
GSK3B	5,827	7,074	-1,247	0,442	9	18	2,822	391
FOXO1	6,653	6,074	0,5794	0,442	9	18	1,311	391
AKT1	3,376	8,006	-4,63	0,442	9	18	10,48	391
AKT2	6,081	7,848	-1,767	0,4463	9	17	3,959	391
AKT3	13,58	9,819	3,757	0,4565	9	15	8,231	391
MAPK3	5,013	9,114	-4,101	0,4463	9	17	9,188	391
MAPK1	4,867	7,075	-2,208	0,442	9	18	4,995	391
HIF1A	3,972	5,559	-1,587	0,442	9	18	3,591	391
PFKL	4,985	9,055	-4,07	0,442	9	18	9,208	391
ACACA	7,023	9,168	-2,145	0,4463	9	17	4,806	391
ACACB	8,327	9,398	-1,071	0,4511	9	16	2,373	391
HMGCL	6,24	8,021	-1,781	0,442	9	18	4,029	391
BDH1	7,68	8,067	-0,3875	0,442	9	18	0,8768	391
LDHA	1,503	3,225	-1,722	0,442	9	18	3,895	391
HK2	11,89	7,901	3,988	0,442	9	18	9,022	391

Table S12. Values of test statistics of protein expression analyses of GSK3A (Figure 4A). P values of 0.05 or less were considered significant (*** $p \leq 0.001$, **** $p < 0.0001$).

**Number of families**	1							
**Number of comparisons per family**	10							
**Alpha**	0,05							
**Tukey's multiple comparisons test**	**Mean Diff,**	**95,00% CI of diff,**	**Significant?**	**Summary**	**Adjusted P Value**			
Column A vs. Column B	0,1621	-6,337 to 6,662	No	ns	>0,9999	A-B		
Column A vs. PHC	0,1605	-6,094 to 6,415	No	ns	>0,9999	A-C		
Column A vs. HepG2	-15,62	-23,02 to -8,224	Yes	****	<0,0001	A-D		
Column A vs. Huh7	-14,62	-22,02 to -7,221	Yes	***	0,0001	A-E		
Column B vs. PHC	-0,00162	-5,869 to 5,865	No	ns	>0,9999	B-C		
Column B vs. HepG2	-15,79	-22,86 to -8,710	Yes	****	<0,0001	B-D		
Column B vs. Huh7	-14,78	-21,86 to -7,708	Yes	****	<0,0001	B-E		
PHC vs. HepG2	-15,78	-22,64 to -8,933	Yes	****	<0,0001	C-D		
PHC vs. Huh7	-14,78	-21,63 to -7,931	Yes	****	<0,0001	C-E		
HepG2 vs. Huh7	1,003	-6,908 to 8,914	No	ns	0,9947	D-E		
**Test details**	**Mean 1**	**Mean 2**	**Mean Diff,**	**SE of diff,**	**n1**	**n2**	**q**	**DF**
Column A vs. Column B	0,346	0,1838	0,1621	2,121	4	5	0,1081	16
Column A vs. PHC	0,346	0,1855	0,1605	2,041	4	6	0,1112	16
Column A vs. HepG2	0,346	15,97	-15,62	2,415	4	3	9,148	16
Column A vs. Huh7	0,346	14,97	-14,62	2,415	4	3	8,561	16
Column B vs. PHC	0,1838	0,1855	-0,00162	1,915	5	6	0,001196	16
Column B vs. HepG2	0,1838	15,97	-15,79	2,31	5	3	9,666	16
Column B vs. Huh7	0,1838	14,97	-14,78	2,31	5	3	9,052	16
PHC vs. HepG2	0,1855	15,97	-15,78	2,236	6	3	9,982	16
PHC vs. Huh7	0,1855	14,97	-14,78	2,236	6	3	9,348	16
HepG2 vs. Huh7	15,97	14,97	1,003	2,582	3	3	0,5492	16

Table S13. Values of test statistics of protein expression analyses of PFKL (Figure 4B). P values of 0.05 or less were considered significant (** $p \leq 0.01$).

**Number of families**	1							
**Number of comparisons per family**	10							
**Alpha**	0,05							
**Tukey's multiple comparisons test**	**Mean Diff,**	**95,00% CI of diff,**	**Significant?**	**Summary**	**Adjusted P Value**			
Column A vs. Column B	0,8009	0,2591 to 1,343	Yes	**	0,0027	A-B		
Column A vs. PHC	0,7679	0,2466 to 1,289	Yes	**	0,0028	A-C		
Column A vs. HepG2	-0,0427	-0,6596 to 0,5742	No	ns	0,9995	A-D		
Column A vs. Huh7	0,2952	-0,3217 to 0,9121	No	ns	0,5972	A-E		
Column B vs. PHC	-0,03299	-0,5221 to 0,4561	No	ns	0,9995	B-C		
Column B vs. HepG2	-0,8436	-1,433 to -0,2538	Yes	**	0,0037	B-D		
Column B vs. Huh7	-0,5058	-1,096 to 0,08411	No	ns	0,112	B-E		
PHC vs. HepG2	-0,8106	-1,382 to -0,2395	Yes	**	0,0039	C-D		
PHC vs. Huh7	-0,4728	-1,044 to 0,09836	No	ns	0,1313	C-E		
HepG2 vs. Huh7	0,3379	-0,3216 to 0,9974	No	ns	0,5358	D-E		
**Test details**	**Mean 1**	**Mean 2**	**Mean Diff,**	**SE of diff,**	**n1**	**n2**	**q**	**DF**
Column A vs. Column B	1,158	0,357	0,8009	0,1769	4	5	6,405	16
Column A vs. PHC	1,158	0,39	0,7679	0,1702	4	6	6,382	16
Column A vs. HepG2	1,158	1,201	-0,0427	0,2014	4	3	0,2999	16
Column A vs. Huh7	1,158	0,8628	0,2952	0,2014	4	3	2,073	16
Column B vs. PHC	0,357	0,39	-0,03299	0,1596	5	6	0,2922	16
Column B vs. HepG2	0,357	1,201	-0,8436	0,1925	5	3	6,197	16
Column B vs. Huh7	0,357	0,8628	-0,5058	0,1925	5	3	3,715	16
PHC vs. HepG2	0,39	1,201	-0,8106	0,1864	6	3	6,15	16
PHC vs. Huh7	0,39	0,8628	-0,4728	0,1864	6	3	3,586	16
HepG2 vs. Huh7	1,201	0,8628	0,3379	0,2153	3	3	2,22	16

Table S14. Values of test statistics of protein expression analyses of HK2 (Figure 4C). P values of 0.05 or less were considered significant (** $p \leq 0.01$, **** $p < 0.0001$).

**Number of families**	1							
**Number of comparisons per family**	10							
**Alpha**	0,05							
**Tukey's multiple comparisons test**	**Mean Diff,**	**95,00% CI of diff,**	**Significant?**	**Summary**	**Adjusted P Value**			
Column A vs. Column B	0,2123	-0,5350 to 0,9595	No	ns	0,9036	A-B		
Column A vs. PHC	0,2053	-0,5138 to 0,9243	No	ns	0,9021	A-C		
Column A vs. HepG2	-5,494	-6,345 to -4,644	Yes	****	<0,0001	A-D		
Column A vs. Huh7	-6,776	-7,626 to -5,925	Yes	****	<0,0001	A-E		
Column B vs. PHC	-0,006988	-0,6815 to 0,6675	No	ns	>0,9999	B-C		
Column B vs. HepG2	-5,707	-6,520 to -4,893	Yes	****	<0,0001	B-D		
Column B vs. Huh7	-6,988	-7,801 to -6,174	Yes	****	<0,0001	B-E		
PHC vs. HepG2	-5,7	-6,487 to -4,912	Yes	****	<0,0001	C-D		
PHC vs. Huh7	-6,981	-7,769 to -6,193	Yes	****	<0,0001	C-E		
HepG2 vs. Huh7	-1,281	-2,191 to -0,3716	Yes	**	0,0042	D-E		
**Test details**	**Mean 1**	**Mean 2**	**Mean Diff,**	**SE of diff,**	**n1**	**n2**	**q**	**DF**
Column A vs. Column B	0,2533	0,04102	0,2123	0,2439	4	5	1,231	16
Column A vs. PHC	0,2533	0,04801	0,2053	0,2347	4	6	1,237	16
Column A vs. HepG2	0,2533	5,748	-5,494	0,2777	4	3	27,98	16
Column A vs. Huh7	0,2533	7,029	-6,776	0,2777	4	3	34,51	16
Column B vs. PHC	0,04102	0,04801	-0,006988	0,2202	5	6	0,04489	16
Column B vs. HepG2	0,04102	5,748	-5,707	0,2655	5	3	30,39	16
Column B vs. Huh7	0,04102	7,029	-6,988	0,2655	5	3	37,22	16
PHC vs. HepG2	0,04801	5,748	-5,7	0,2571	6	3	31,35	16
PHC vs. Huh7	0,04801	7,029	-6,981	0,2571	6	3	38,4	16
HepG2 vs. Huh7	5,748	7,029	-1,281	0,2969	3	3	6,103	16

Table S15. Values of test statistics of protein expression analyses of ACACB (Figure 4D). P values of 0.05 or less were considered significant (* $p \leq 0.05$, ** $p \leq 0.01$, *** $p \leq 0.001$).

**Number of families**	1							
**Number of comparisons per family**	10							
**Alpha**	0,05							
**Tukey's multiple comparisons test**	**Mean Diff,**	**95,00% CI of diff,**	**Significant?**	**Summary**	**Adjusted P Value**			
Column A vs. Column B	0,3049	-0,2014 to 0,8112	No	ns	0,3836	A-B		
Column A vs. PHC	0,9124	0,4253 to 1,400	Yes	***	0,0003	A-C		
Column A vs. HepG2	0,9461	0,3696 to 1,523	Yes	**	0,001	A-D		
Column A vs. Huh7	0,9551	0,3787 to 1,532	Yes	***	0,0009	A-E		
Column B vs. PHC	0,6075	0,1505 to 1,065	Yes	**	0,0068	B-C		
Column B vs. HepG2	0,6412	0,08999 to 1,192	Yes	*	0,0188	B-D		
Column B vs. Huh7	0,6502	0,09904 to 1,201	Yes	*	0,017	B-E		
PHC vs. HepG2	0,03365	-0,5000 to 0,5673	No	ns	0,9997	C-D		
PHC vs. Huh7	0,0427	-0,4910 to 0,5764	No	ns	0,9991	C-E		
HepG2 vs. Huh7	0,00905	-0,6072 to 0,6253	No	ns	>0,9999	D-E		
**Test details**	**Mean 1**	**Mean 2**	**Mean Diff,**	**SE of diff,**	**n1**	**n2**	**q**	**DF**
Column A vs. Column B	1,031	0,7262	0,3049	0,1653	4	5	2,609	16
Column A vs. PHC	1,031	0,1187	0,9124	0,159	4	6	8,115	16
Column A vs. HepG2	1,031	0,08506	0,9461	0,1882	4	3	7,111	16
Column A vs. Huh7	1,031	0,07601	0,9551	0,1882	4	3	7,179	16
Column B vs. PHC	0,7262	0,1187	0,6075	0,1492	5	6	5,76	16
Column B vs. HepG2	0,7262	0,08506	0,6412	0,1799	5	3	5,04	16
Column B vs. Huh7	0,7262	0,07601	0,6502	0,1799	5	3	5,111	16
PHC vs. HepG2	0,1187	0,08506	0,03365	0,1742	6	3	0,2732	16
PHC vs. Huh7	0,1187	0,07601	0,0427	0,1742	6	3	0,3467	16
HepG2 vs. Huh7	0,08506	0,07601	0,00905	0,2011	3	3	0,06363	16

Table S16. Values of test statistics of protein expression analyses of BDH1 (Figure 4E). P values of 0.05 or less were considered significant (* $p \leq 0.05$).

**Number of families**	1							
**Number of comparisons per family**	10							
**Alpha**	0,05							
**Tukey's multiple comparisons test**	**Mean Diff,**	**diff,**	**Significant?**	**Summary**	**Adjusted P Value**			
Column A vs. Column B	-4,165	-7,619 to -0,7100	Yes	*	0,0146	A-B		
Column A vs. PHC	-2,149	-5,473 to 1,175	No	ns	0,3184	A-C		
Column A vs. HepG2	-1,109	-5,042 to 2,824	No	ns	0,9059	A-D		
Column A vs. Huh7	-0,995	-4,928 to 2,938	No	ns	0,9342	A-E		
Column B vs. PHC	2,016	-1,103 to 5,134	No	ns	0,3184	B-C		
Column B vs. HepG2	3,056	-0,7052 to 6,817	No	ns	0,1424	B-D		
Column B vs. Huh7	3,17	-0,5913 to 6,931	No	ns	0,1212	B-E		
PHC vs. HepG2	1,04	-2,601 to 4,681	No	ns	0,9019	C-D		
PHC vs. Huh7	1,154	-2,488 to 4,795	No	ns	0,8643	C-E		
HepG2 vs. Huh7	0,1139	-4,091 to 4,319	No	ns	>0,9999	D-E		
**Test details**	**Mean 1**	**Mean 2**	**Mean Diff,**	**SE of diff,**	**n1**	**n2**	**q**	**DF**
Column A vs. Column B	0,456	4,621	-4,165	1,128	4	5	5,223	16
Column A vs. PHC	0,456	2,605	-2,149	1,085	4	6	2,801	16
Column A vs. HepG2	0,456	1,565	-1,109	1,284	4	3	1,222	16
Column A vs. Huh7	0,456	1,451	-0,995	1,284	4	3	1,096	16
Column B vs. PHC	4,621	2,605	2,016	1,018	5	6	2,801	16
Column B vs. HepG2	4,621	1,565	3,056	1,228	5	3	3,52	16
Column B vs. Huh7	4,621	1,451	3,17	1,228	5	3	3,651	16
PHC vs. HepG2	2,605	1,565	1,04	1,189	6	3	1,237	16
PHC vs. Huh7	2,605	1,451	1,154	1,189	6	3	1,373	16
HepG2 vs. Huh7	1,565	1,451	0,1139	1,372	3	3	0,1174	16

Table S17. Values of test statistics of protein expression analyses of LDHA (Figure 4F). P values of 0.05 or less were considered significant (* $p \leq 0.05$, ** $p \leq 0.01$, *** $p \leq 0.001$).

**Number of families**	1							
**Number of comparisons per family**	10							
**Alpha**	0,05							
**Tukey's multiple comparisons test**	**Mean Diff,**	**95,00% CI of diff,**	**Significant?**	**Summary**	**Adjusted P Value**			
Column A vs. Column B	-0,9055	-5,486 to 3,675	No	ns	0,9712	A-B		
Column A vs. PHC	1,831	-2,351 to 6,013	No	ns	0,665	A-C		
Column A vs. HepG2	-7,186	-12,13 to -2,238	Yes	**	0,0034	A-D		
Column A vs. Huh7	-4,41	-9,358 to 0,5377	No	ns	0,0922	A-E		
Column B vs. PHC	2,736	-1,445 to 6,918	No	ns	0,3031	B-C		
Column B vs. HepG2	-6,281	-11,23 to -1,333	Yes	*	0,0102	B-D		
Column B vs. Huh7	-3,505	-8,453 to 1,443	No	ns	0,2367	B-E		
PHC vs. HepG2	-9,017	-13,60 to -4,437	Yes	***	0,0002	C-D		
PHC vs. Huh7	-6,241	-10,82 to -1,660	Yes	**	0,0058	C-E		
HepG2 vs. Huh7	2,776	-2,513 to 8,066	No	ns	0,5075	D-E		
**Test details**	**Mean 1**	**Mean 2**	**Mean Diff,**	**SE of diff,**	**n1**	**n2**	**q**	**DF**
Column A vs. Column B	3,4	4,305	-0,9055	1,484	4	4	0,8632	15
Column A vs. PHC	3,4	1,569	1,831	1,354	4	6	1,912	15
Column A vs. HepG2	3,4	10,59	-7,186	1,602	4	3	6,343	15
Column A vs. Huh7	3,4	7,81	-4,41	1,602	4	3	3,892	15
Column B vs. PHC	4,305	1,569	2,736	1,354	4	6	2,858	15
Column B vs. HepG2	4,305	10,59	-6,281	1,602	4	3	5,543	15
Column B vs. Huh7	4,305	7,81	-3,505	1,602	4	3	3,093	15
PHC vs. HepG2	1,569	10,59	-9,017	1,484	6	3	8,596	15
PHC vs. Huh7	1,569	7,81	-6,241	1,484	6	3	5,95	15
HepG2 vs. Huh7	10,59	7,81	2,776	1,713	3	3	2,292	15

Table S18. Values of test statistics of glucose consumption analyses (Figure 5A).

**Number of families**	1							
**Number of comparisons per family**	6							
**Alpha**	0,05							
**Tukey's multiple comparisons test**	**Mean Diff,**	**95,00% CI of diff,**	**Significant?**	**Summary**	**Adjusted P Value**			
Column A vs. Column B	0,6793	-0,7822 to 2,141	No	ns	0,5147	A-B		
Column A vs. HepG2	0,004667	-1,574 to 1,583	No	ns	>0,9999	A-C		
Column A vs. Huh7	-0,008	-1,587 to 1,571	No	ns	>0,9999	A-D		
Column B vs. HepG2	-0,6746	-2,253 to 0,9040	No	ns	0,579	B-C		
Column B vs. Huh7	-0,6873	-2,266 to 0,8913	No	ns	0,565	B-D		
HepG2 vs. Huh7	-0,01267	-1,700 to 1,675	No	ns	>0,9999	C-D		
**Test details**	**Mean 1**	**Mean 2**	**Mean Diff,**	**SE of diff,**	**n1**	**n2**	**q**	**DF**
Column A vs. Column B	0,03	-0,6493	0,6793	0,4777	4	4	2,011	10
Column A vs. HepG2	0,03	0,02533	0,004667	0,516	4	3	0,01279	10
Column A vs. Huh7	0,03	0,038	-0,008	0,516	4	3	0,02193	10
Column B vs. HepG2	-0,6493	0,02533	-0,6746	0,516	4	3	1,849	10
Column B vs. Huh7	-0,6493	0,038	-0,6873	0,516	4	3	1,884	10
HepG2 vs. Huh7	0,02533	0,038	-0,01267	0,5516	3	3	0,03247	10

Table S19. Values of test statistics of glycogen storage analyses (Figure 5B). P values of 0.05 or less were considered significant (*** $p \leq 0.001$, **** $p < 0.0001$).

**Number of families**	1							
**Number of comparisons per family**	6							
**Alpha**	0,05							
**Tukey's multiple comparisons test**	**Mean Diff,**	**95,00% CI of diff,**	**Significant?**	**Summary**	**Adjusted P Value**			
Column A vs. Column B	844,1	537,7 to 1150	Yes	****	<0,0001	A-B		
Column A vs. HepG2	720,3	389,4 to 1051	Yes	***	0,0003	A-C		
Column A vs. Huh7	720,5	389,6 to 1051	Yes	***	0,0003	A-D		
Column B vs. HepG2	-123,8	-454,7 to 207,1	No	ns	0,6721	B-C		
Column B vs. Huh7	-123,6	-454,4 to 207,3	No	ns	0,6735	B-D		
HepG2 vs. Huh7	0,255	-353,5 to 354,0	No	ns	>0,9999	C-D		
**Test details**	**Mean 1**	**Mean 2**	**Mean Diff,**	**SE of diff,**	**n1**	**n2**	**q**	**DF**
Column A vs. Column B	846,2	2,104	844,1	100,1	4	4	11,92	10
Column A vs. HepG2	846,2	125,9	720,3	108,2	4	3	9,418	10
Column A vs. Huh7	846,2	125,7	720,5	108,2	4	3	9,422	10
Column B vs. HepG2	2,104	125,9	-123,8	108,2	4	3	1,619	10
Column B vs. Huh7	2,104	125,7	-123,6	108,2	4	3	1,616	10
HepG2 vs. Huh7	125,9	125,7	0,255	115,6	3	3	0,003119	10

Table S20. Values of test statistics of lactate consumption analyses (Figure 5C). P values of 0.05 or less were considered significant (**** $p < 0.0001$).

**Number of families**	1							
**Number of comparisons per family**	6							
**Alpha**	0,05							
**Tukey's multiple comparisons test**	**Mean Diff,**	**95,00% CI of diff,**	**Significant?**	**Summary**	**Adjusted P Value**			
Column A vs. Column B	-0,145	-0,1993 to -0,09066	Yes	****	<0,0001	A-B		
Column A vs. HepG2	-0,0005	-0,05919 to 0,05819	No	ns	>0,9999	A-C		
Column A vs. Huh7	0,009833	-0,04886 to 0,06852	No	ns	0,9542	A-D		
Column B vs. HepG2	0,1445	0,08581 to 0,2032	Yes	****	<0,0001	B-C		
Column B vs. Huh7	0,1548	0,09614 to 0,2135	Yes	****	<0,0001	B-D		
HepG2 vs. Huh7	0,01033	-0,05241 to 0,07307	No	ns	0,9563	C-D		
**Test details**	**Mean 1**	**Mean 2**	**Mean Diff,**	**SE of diff,**	**n1**	**n2**	**q**	**DF**
Column A vs. Column B	-0,0185	0,1265	-0,145	0,01776	4	4	11,55	10
Column A vs. HepG2	-0,0185	-0,018	-0,0005	0,01918	4	3	0,03686	10
Column A vs. Huh7	-0,0185	-0,02833	0,009833	0,01918	4	3	0,7249	10
Column B vs. HepG2	0,1265	-0,018	0,1445	0,01918	4	3	10,65	10
Column B vs. Huh7	0,1265	-0,02833	0,1548	0,01918	4	3	11,41	10
HepG2 vs. Huh7	-0,018	-0,02833	0,01033	0,02051	3	3	0,7126	10

Table S21. Values of test statistics of pyruvate analyses (Figure 5D). P values of 0.05 or less were considered significant (** $p \leq 0.01$, *** $p \leq 0.001$, **** $p < 0.0001$).

**Number of families**	1							
**Number of comparisons per family**	6							
**Alpha**	0,05							
**Tukey's multiple comparisons test**	**Mean Diff,**	**95,00% CI of diff,**	**Significant?**	**Summary**	**Adjusted P Value**			
Column A vs. Column B	1,216	0,9006 to 1,532	Yes	****	<0,0001	A-B		
Column A vs. HepG2	0,6777	0,3404 to 1,015	Yes	***	0,0007	A-C		
Column A vs. Huh7	0,519	0,1817 to 0,8563	Yes	**	0,0044	A-D		
Column B vs. HepG2	-0,5384	-0,8539 to -0,2229	Yes	**	0,0022	B-C		
Column B vs. Huh7	-0,6971	-1,013 to -0,3816	Yes	***	0,0003	B-D		
HepG2 vs. Huh7	-0,1587	-0,4959 to 0,1786	No	ns	0,4924	C-D		
**Test details**	**Mean 1**	**Mean 2**	**Mean Diff,**	**SE of diff,**	**n1**	**n2**	**q**	**DF**
Column A vs. Column B	1,313	0,09725	1,216	0,1011	3	4	17,02	9
Column A vs. HepG2	1,313	0,6357	0,6777	0,108	3	3	8,871	9
Column A vs. Huh7	1,313	0,7943	0,519	0,108	3	3	6,794	9
Column B vs. HepG2	0,09725	0,6357	-0,5384	0,1011	4	3	7,535	9
Column B vs. Huh7	0,09725	0,7943	-0,6971	0,1011	4	3	9,755	9
HepG2 vs. Huh7	0,6357	0,7943	-0,1587	0,108	3	3	2,077	9

Table S22. Values of test statistics of ketone bodies analyses (Figure 5E). P values of 0.05 or less were considered significant (** $p \leq 0.01$).

**Number of families**	1							
**Number of comparisons per family**	6							
**Alpha**	0,05							
**Tukey's multiple comparisons test**	**Mean Diff,**	**95,00% CI of diff,**	**Significant?**	**Summary**	**Adjusted P Value**			
Column A vs. Column B	-0,02258	-0,04912 to 0,003952	No	ns	0,1004	A-B		
Column A vs. HepG2	0,019	-0,009368 to 0,04737	No	ns	0,2268	A-C		
Column A vs. Huh7	0,018	-0,01037 to 0,04637	No	ns	0,2633	A-D		
Column B vs. HepG2	0,04158	0,01505 to 0,06812	Yes	**	0,0039	B-C		
Column B vs. Huh7	0,04058	0,01405 to 0,06712	Yes	**	0,0046	B-D		
HepG2 vs. Huh7	-0,001	-0,02937 to 0,02737	No	ns	0,9995	C-D		
**Test details**	**Mean 1**	**Mean 2**	**Mean Diff,**	**SE of diff,**	**n1**	**n2**	**q**	**DF**
Column A vs. Column B	0,02167	0,04425	-0,02258	0,0085	3	4	3,757	9
Column A vs. HepG2	0,02167	0,002667	0,019	0,009087	3	3	2,957	9
Column A vs. Huh7	0,02167	0,003667	0,018	0,009087	3	3	2,801	9
Column B vs. HepG2	0,04425	0,002667	0,04158	0,0085	4	3	6,918	9
Column B vs. Huh7	0,04425	0,003667	0,04058	0,0085	4	3	6,752	9
HepG2 vs. Huh7	0,002667	0,003667	-0,001	0,009087	3	3	0,1556	9

Table S23. Values of test statistics of lipid storage analyses (Figure 5F).

**Number of families**	1							
**Number of comparisons per family**	6							
**Alpha**	0,05							
**Tukey's multiple comparisons test**	**Mean Diff,**	**95,00% CI of diff,**	**Significant?**	**Summary**	**Adjusted P Value**			
Column A vs. Column B	-0,1142	-0,3435 to 0,1152	No	ns	0,4479	A-B		
Column A vs. HepG2	0,031	-0,2142 to 0,2762	No	ns	0,9779	A-C		
Column A vs. Huh7	0,027	-0,2182 to 0,2722	No	ns	0,9851	A-D		
Column B vs. HepG2	0,1452	-0,08416 to 0,3745	No	ns	0,265	B-C		
Column B vs. Huh7	0,1412	-0,08816 to 0,3705	No	ns	0,2848	B-D		
HepG2 vs. Huh7	-0,004	-0,2492 to 0,2412	No	ns	>0,9999	C-D		
**Test details**	**Mean 1**	**Mean 2**	**Mean Diff,**	**SE of diff,**	**n1**	**n2**	**q**	**DF**
Column A vs. Column B	0,03533	0,1495	-0,1142	0,07346	3	4	2,198	9
Column A vs. HepG2	0,03533	0,004333	0,031	0,07853	3	3	0,5582	9
Column A vs. Huh7	0,03533	0,008333	0,027	0,07853	3	3	0,4862	9
Column B vs. HepG2	0,1495	0,004333	0,1452	0,07346	4	3	2,795	9
Column B vs. Huh7	0,1495	0,008333	0,1412	0,07346	4	3	2,718	9
HepG2 vs. Huh7	0,004333	0,008333	-0,004	0,07853	3	3	0,07203	9
